# A Dereplication and Bioguided Discovery Approach to Reveal New Compounds from a Marine-Derived Fungus *Stilbella fimetaria*

**DOI:** 10.3390/md15080253

**Published:** 2017-08-13

**Authors:** Sara Kildgaard, Karolina Subko, Emma Phillips, Violaine Goidts, Mercedes de la Cruz, Caridad Díaz, Charlotte H. Gotfredsen, Birgitte Andersen, Jens C. Frisvad, Kristian F. Nielsen, Thomas O. Larsen

**Affiliations:** 1 DTU Bioengineering, Technical University of Denmark, Søltofts Plads 221, DK-2800 Kgs. Lyngby, Denmark; sarki@bio.dtu.dk (S.K.); karosu@dtu.dk (K.S.); ba@bio.dtu.dk (B.A.); jcf@bio.dtu.dk (J.C.F.); kfn@bio.dtu.dk (K.F.N.); 2 German Cancer Research Center, Brain Tumor Translational Targets, Im Neuenheimer Feld 580, D-69120 Heidelberg, Germany; e.phillips@dkfz-heidelberg.de (E.P.); v.goidts@dkfz-heidelberg.de (V.G.); 3 Fundación MEDINA, Av del Conocimiento, 34, 18100 Armilla, Granada, Spain; mercedes.delacruz@medinaandalucia.es (M.d.l.C.); caridad.diaz@medinaandalucia.es (C.D.); 4 Department of Chemistry, Technical University of Denmark, Kemitorvet, Building 207, DK-2800 Kgs. Lyngby, Denmark; chg@kemi.dtu.dk

**Keywords:** bioguided-discovery, dereplication, cytotoxicity, antifungal, MS/HRMS, marine-derived, pimarane-type diterpenoids, ilicicolin H

## Abstract

A marine-derived *Stilbella fimetaria* fungal strain was screened for new bioactive compounds based on two different approaches: (i) bio-guided approach using cytotoxicity and antimicrobial bioassays; and (ii) dereplication based approach using liquid chromatography with both diode array detection and high resolution mass spectrometry. This led to the discovery of several bioactive compound families with different biosynthetic origins, including pimarane-type diterpenoids and hybrid polyketide-non ribosomal peptide derived compounds. Prefractionation before bioassay screening proved to be a great aid in the dereplication process, since separate fractions displaying different bioactivities allowed a quick tentative identification of known antimicrobial compounds and of potential new analogues. A new pimarane-type diterpene, myrocin F, was discovered in trace amounts and displayed cytotoxicity towards various cancer cell lines. Further media optimization led to increased production followed by the purification and bioactivity screening of several new and known pimarane-type diterpenoids. A known broad-spectrum antifungal compound, ilicicolin H, was purified along with two new analogues, hydroxyl-ilicicolin H and ilicicolin I, and their antifungal activity was evaluated.

## 1. Introduction

With the ocean covering almost two thirds of the Earth’s surface area, the marine environment offers a great diversity of microorganisms and thereby a promising potential for new bioactive natural products displaying unique chemical scaffolds [1,2,3]. Fungal strains isolated from the marine environment have attracted increased attention due to the discovery of several secondary metabolites rich in biological activity [1,2,3,4]. The majority of fungal strains have been isolated from sources such as algae, sponges, and mangrove habitats [1] with deep sea sediments emerging as a new niche of potentially interesting compounds [1,5]. It is under much debate, however, what the real origin of these fungal strains is; being true marine or opportunistic strains adapted to the marine environment [4]. This is due to the fact that many fungal strains are isolated from intertidal zones and mangrove habitats and thereby not likely true marine habitats [4,6,7,8]. The most common secondary metabolite producing species come from *Aspergillus* and *Penicillium*, with only few belonging to the well-documented lineage of marine fungi [4,6,7,8]. That said, the origin of these marine-derived fungal strains, whether true marine or opportunistic, may not be as critical when it comes to drug discovery if the opportunistic strains produce new bioactive compounds not found in their terrestrial counterparts.

Dereplication is an essential step in natural product (NP) discovery to prevent re-isolation and re-characterization of known bioactive compounds. It is especially important in primary bioactivity screening, where the target is often non-selective and there is a high chance of rediscovering general cytotoxic compounds. This is due to a great number of highly bioactive compounds being observed across the fungal kingdom of which several are found in multiple fungal species [9,10,11]. One dereplication approach is based on ultra-high performance liquid chromatography-diode array detection-quadrupole time of flight mass spectrometry (UHPLC-DAD-QTOFMS) and database searching [10,12,13]. This can be combined with auto tandem high resolution mass spectrometry (MS/HRMS) and use of a MS/HRMS library, which has been shown to be a robust and effective way of tentatively identifying known bioactive compounds on a given instrument [9,11]. The MS/HRMS library may serve as a database of compounds for targeted dereplication, matching peaks in the unknown spectrum against the library spectrum and vice versa, or to identify compounds sharing similar fragment ions, but that do not share the same molecular formula [11]. Another dereplication strategy based on MS/MS involves molecular networking proposed by the Dorrestein/Bandeira labs [14,15,16], where a pairwise comparison of MS/MS spectra results in clustered networks of structurally related compounds. Early integration of the MS/MS networking approach and bioassay data has been shown to enable the targeted discovery of new bioactive compounds [17]. However, a limiting factor is the lack of back-integration of raw data to find the corresponding full scan data and retention times resulting in detailed analysis being time consuming.

Pre-fractionation serves as another highly valuable step in NP discovery for the success of both the initial dereplication and bioactivity screening. This is because metabolites present in minor amounts may go undetected, their activity being masked or interfered with by major components in a complex crude extract [18,19,20]. Wyeth [19] and Appleton et al. [18] reported that primary bioactivity screening of pre-fractionated crude samples showed that the bioactivity was masked in up to 80% of the cases with no activity being observed for the original crude extracts, but only for the fractions. Meanwhile, up to 13% of the crude samples lost their activity upon fractionation [18,19], meaning that it can be advantageous to screen both the crude extract and fractions in the primary assay. In addition to using traditional reversed phase (RP) chromatography for pre-fractionation, orthogonal purification strategies such as Explorative Solid-Phase Extraction (E-SPE) can be used to facilitate the removal or reduction of co-eluting interferences [21]. Pre-fractionation can aid the discovery of new compounds or activities which would have otherwise been missed either due to: (1) the crude extract containing more than one compound responsible for the observed activity; (2) a single compound displaying multiple activities, or (3) several compounds displaying various activities.

In this paper, we describe a combined bioassay-guided and dereplication based discovery approach for a marine-derived fungus *Stilbella fimetaria* IBT 28361 using cytotoxicity and antimicrobial screening assays and UHPLC-DAD-QTOFMS with MS/HRMS in combination with our in-house MS/HRMS library [9]. This method led to the discovery of different bioactive compound families in *Stilbella fimetaria,* including pimarane-type diterpenoids and hybrid polyketide-non ribosomal peptides belonging to the ilicicolin H family. To the best of our knowledge, the latter has not previously been obtained from the genus *Stilbella*. New and known compounds of both families were isolated and elucidated by nuclear magnetic resonance (NMR) spectroscopy and their cytotoxicity and antimicrobial activities evaluated. The outcome of the primary bioassay screening on both the crude extracts and their fractions assisted in the dereplication of the crude extract allowing for a quick tentative identification of known antimicrobial compounds and potential new bioactive analogues.

## 2. Results and Discussion

**Figure 1:**
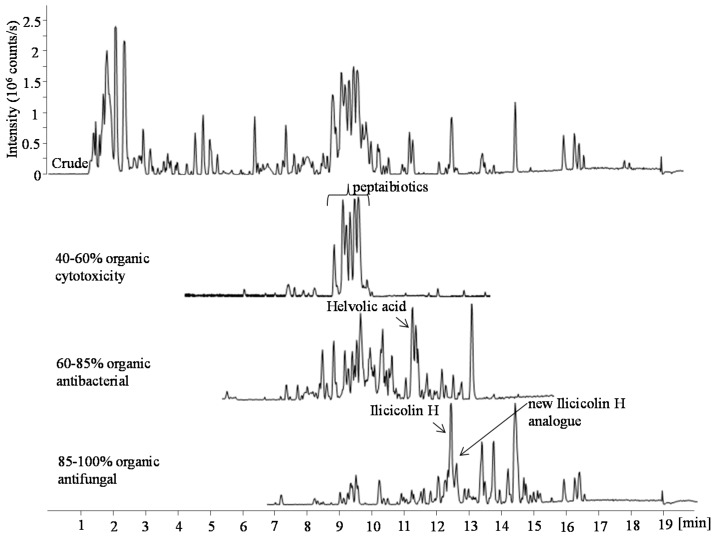
Base peak chromatograms (BPC) of the EtOAc crude extract and three bioactive fractions (ranging from 40% to 100% organic) in positive electrospray ionization (ESI) mode. The fractions were obtained by RP flash chromatography with a gradient of MeCN and water going from 15% to 100% MeCN. In the bioactive fractions the marked peaks indicate the tentatively identified peptaiboitics, helvolic acid, ilicicolin H, and a potential new ilicicolin H analogue.

Bioactivity-guided purification was performed using cytotoxicity and antimicrobial bioassays on the marine-derived fungus *Stilbella fimetaria* IBT 28361 isolated from a seawater sample off the coast of the island Fanoe, Western part of Denmark. The fungus was cultivated in small cultivation on yeast extract sucrose agar (YES) and czapek yeast extract agar (CYA) plates. The YES and CYA plates were combined and extracted together with EtOAc containing 1% formic acid (FA). An EtOAC extract of plates from both YES and CYA media were chosen in order to increase the spectrum of compounds produced by the fungus. The EtOAc crude extract was fractionated by RP flash chromatography with a gradient of acetonitrile (MeCN) and water going from 15% to 100% MeCN into six fractions and both the crude extract and the six fractions were subsequently evaluated for their cytotoxicity and antimicrobial activity. No activity was observed for the screening of the crude extract on its own, whereas the fourth, fifth, and sixth flash fractions (ranging from 40% to 100% organic) displayed cytotoxic, antibacterial, and antifungal activities, respectively (Figure 1). Dereplication of the separate bioactive fractions using UHPLC-DAD-QTOFMS allowed a quick tentative identification of several bioactive compound families likely responsible for the observed activities (Figure 1).

Different further fungal cultivations (1–3, see below) were prepared in order to purify potential new compounds and confirm the bioactivity of the known compounds in the applied bioassays. The identity of the cytotoxic compounds and their analogues were obtained using bio-guided isolation on large scale cultivation YES media (Cultivation 1). YES media was chosen as it displayed weak activity when crude extracts for one YES and one CYA plate were tested separately. Further media optimization was performed to find the optimal growth conditions for potential cytotoxic compounds otherwise only present in trace amounts on YES media and in order to circumvent a group of co-eluting peptaibiotics (Cultivation 2, rice media incubated for 10 days at 25 °C in the dark) [Section 2.1]. The known antibacterial nortriterpenoid, helvolic acid [22,23,24] was identified as one of the main components in the fraction displaying antibacterial activity against methicillin-resistant *Staphylococcus aureus* (MRSA) reported by Kildgaard et al. [11]. This was done using an in-house MS/HRMS library and comparison of its retention time to a standard from our compound library and suggested to be the compound exhibiting the observed activity. In this study, the activity of the pure compound against MRSA was confirmed, MIC_90_ < 0.25 µg/mL (1D NMR data shown in Figures S9–S11). The compound responsible for the antifungal activity was tentatively identified as the broad-spectrum antifungal metabolite, ilicicolin H [25,26,27,28]. Due to the tentative identification of new ilicicolin H analogues with similar retention times to ilicicolin H, it was isolated along with two new analogues on large cultivation rice media incubated for 21 days at 25 °C in the dark (Cultivation 3) [Section 2.2].

### 2.1. Pimarane-Type Diterpenoids Exhibiting Cytotoxicity

**Figure 2:**
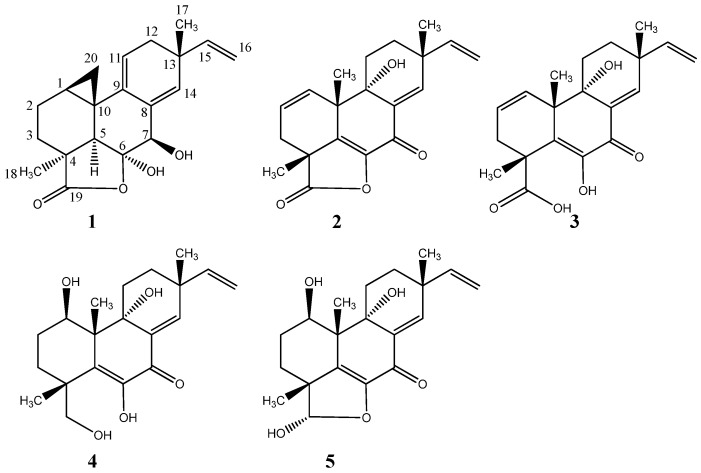
Structures of pimarane diterpenoids; myrocin F (**1**), libertellenone M (**2**), the suggested opened γ-lactone of libertellenone M (**3**), libertellenone C (**4**), and libertellenone E (**5**).

From cultivation 1, the RP flash chromatography fractions (40–60% organic) displayed cytotoxicity against patient derived glioblastoma stem-like cells (GSCs). The observed cytotoxicity was comparable to that of the fraction from the original small scale EtOAc crude extract of the YES and CYA plates combined and extracted together. GSCs were chosen for the study as there is an acute need for novel therapeutics targeting this tumor subpopulation as they exhibit resistance to the current standard therapy for glioblastoma [29]. UHPLC-DAD-QTOFMS-MS/HRMS and use of an in-house MS/HRMS library [11] and The Comprehensive Peptaibiotics Database [30] tentatively identified the major components of the active fractions to be peptaibiotics belonging to the antiamebins family. Antiamebin I [31] was tentatively identified by comparison to its reference standard in the MS/HRMS library reported by Kildgaard et al. [11]. Furthermore, *Stilbella fimetaria* (syn. *S. erythrocephala*) is well-known for its production of antiamebins [32,33]. At a glance, the antiamebins might be suspected as responsible for the observed bioactivity, but a second fractionation revealed the active component to be the compound obscured by the group of co-eluting antiamebins and present only in trace amount in the first fractionation. The molecular formula for the compound was established to be C_20_H_22_O_4_ based on the pseudomolecular ion, [M + H]^+^ of *m*/*z* 329.1745 with an accuracy of 0.6 ppm (HRESITOFMS) and the ultraviolet (UV) spectrum displayed absorption bands at λ_max_ 215 and 270 nm. A NMR spectroscopic analysis of the isolated compound and comparison to the data reported for myrocins A-E [34,35,36,37] allowed for the structural elucidation of a new pimarane-type diterpene, myrocin F (**1**) (Figure 2). ^1^H, ^13^C, HMBC and NOESY data is shown in Table 1.

**Figure 3:**
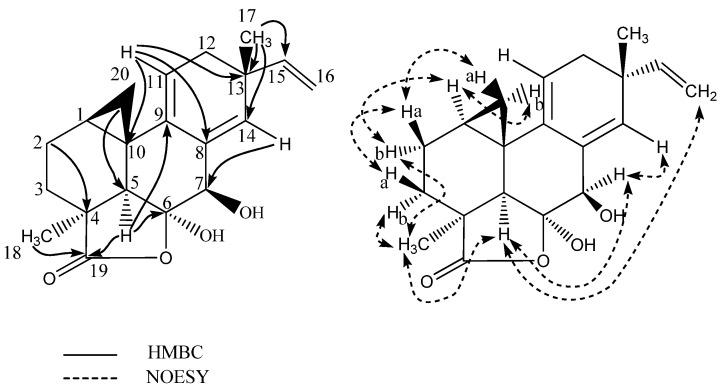
Selected key HMBC and NOESY correlations for myrocin F (**1**).

^1^H-NMR spectrum revealed the presence of six methines (including one oxygenated and three olefinic), five methylenes (including one cyclopropylic and one olefinic), and two singlet methyl groups. The ^13^C-NMR spectrum identified one ester carbonyl group and six quaternary carbon signals (including one oxygenated and two olefinic). The DQF-COSY spectrum defined three spin systems besides the two singlet methyl signals at δ_H_ 1.15 (CH_3_-17) and δ_H_ 1.42 (CH_3_-18). One spin system included the terminal vinyl group with protons at δ_H_ 5.67 (1H, dd, *J* = 17.4, 10.4, H-15), δ_H_ 5.03 (1H, dd, *J* = 17.4, 1.5, H-16a), and δ_H_ 4.89 (1H, dd, *J* = 10.4, 1.5, H-16b). The second spin system included the olefinc methine at δ_H_ 5.24 (CH-11) and the enantiotopic methylene at δ_H_ 2.19 (CH_2_-12). The third spin system included the diastereotropic methylenes at δ_H_ 1.74/1.44 (CH_2_-3) and δ_H_ 1.81/1.78 (CH_2_-2) and cyclopropylic protons (as indicated by the characteristic upfield chemical shift and coupling pattern of H-20) at δ_H_ 1.63 (H-1), δ_H_ 0.85 (1H, t, *J* = 5.2, H-20a), and δ_H_ 0.25 (1H, dd, *J* = 8.2, 5.7, H-20b). The connection of these COSY spin systems and assignment of remaining signals and quaternary carbons was done by analysis of the HMBC spectrum obtaining the long range H-C correlations. The important HMBC correlations from H-16 to C-13, H-17 to C-12, C-13, C-14, and C-15, H-11 to C-8, C-10, and C-13, H-14 to C-7, C-9, C-12, C-13, and C-17, H-7 to C-5, C-6, C-8, and C-9, H-5 to C-4, C-6, C-9, C-10, and C-18, H-2 and H-3 to C-4 confirmed the presence of the pimarane-type diterpene structure [36]. The fusion of the lactone ring through C-4 and C-6 and the cyclopropyl ring (C-1-C-20-C-10) to the pimarane skeleton was supported by the important HMBC correlations from H-5, H-3, and H-18 to the ester carbonyl signal at δ_H_ 185.6 (C-19) and from H-20a to C-1, C-5, and C-9 and H-20b to C-2 and C-9 (See Figure 3 for important HMBC correlations). The relative configuration of myrocin F is based on an analysis of the NOESY spectrum with key NOE correlations from H-1 to H-2b and H-5, from H-5 to H-2b, CH_3_-18, H-7, and H-16a, and from CH_3_-18 to H-2b, H-5, and H-3b. Further key NOEs from H-20a to H-2a and H-3a and between the latter two and from H-20b to H-1 and H-11 and between the latter two suggested the cyclopropyl group with the cyclopropylic protons H-20a/H-20b in each direction, CH_3_-17 and the hydroxyl group at C-7 to be on the same face of the molecule and CH_3_-18, H-5, and the vinyl group on the opposite face. The hydroxyl group at C-6 is suggested to be positioned to the latter face based on observed strong NOE correlation between H-7 and H-14 and no correlation between H-7 and CH_3_-18 (See Figure 3). The relative configuration of myrocin F is in agreement with that of previously reported myrocin A-C [34,35] with absolute configuration reported by X-ray diffraction analysis of myrocin C monoacetate [38].

Optimization of media and growth conditions led to the discovery of a highly enriched profile of the pimarane-type diterpenoids when *Stilbella fimetaria* IBT 28361 was grown on rice media for 10 days at 25 °C in the dark (Cultivation 2). This enabled the purification of two new libertellenones obtained from the same fraction, libertellenone M (**2**) and what we propose to be the opened γ-lactone ring of libertellenone M (**3**) that were both present as trace amounts in cultivation 1 (Figure 2). The two known compounds, libertellenone C (**4**) [39] and libertellenone E (**5**) [36] (Figure 2, Figure S31 and Table 2 with NMR Spectroscopic Data, Supplementary information) were isolated from the same extract and identified based on their spectroscopic profiles (1D and 2D NMR, HRMS, MS/HRMS, UV, ${\left[\alpha \right]}_{D}^{20}$). Both compounds have been reported with their absolute configurations, determined by X-ray diffraction analysis for libertellenone E [36]. The BPC of the crude rice extract with extracted ion chromatogram (EIC) identifying the libertellenones is shown in Figure S4, Supplementary information.

**Figure 4:**
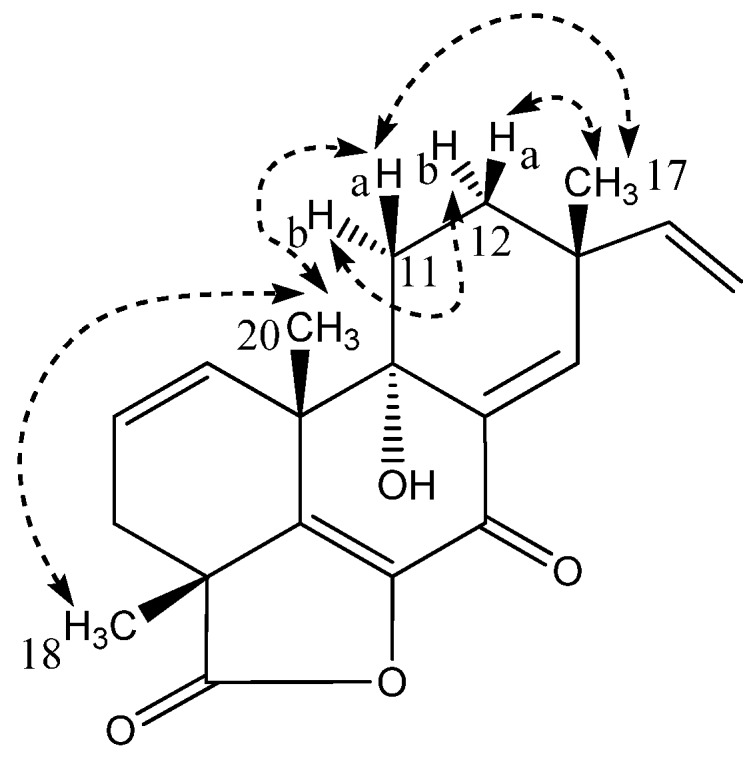
Selected key NOESY correlations for libertellenone M (**2**).

Libertellenone M (**2**) possessed the molecular formula C_20_H_22_O_4_ based on the pseudomolecular ion, [M + H]^+^ of *m*/*z* 327.1592 (accuracy −0.27 ppm). The ^1^H and ^13^C NMR spectra were similar to those of libertellenone C with the exception of the C-1/C-2 double bond with the downfield carbon shifts of δ_C_ 130.7 (C-1) and δ_C_ 127.4 (C-2) compared to δ_C_ 70.1 (C-1) and δ_C_ 29.3 (C-2) and replacement of the ketone at δ_C_ 181.2 (C-19) with the hydroxyl methylene group at δ_C_ 70.2 (opened γ-lactam ring). The planar structure of libertellenone M is the same as that reported for libertellenone G [40] with a different relative configuration suggested based on the NOESY spectra (Figure 4). Key NOEs of compound (**2**) were observed from CH_3_-17 to H-11a and H-12a, from H-11a to H-12a, CH_3_-20 and a weak NOE to CH_3_-18, from CH_3_-20 to H-12a and CH_3_-18, and from H-11b to H-12b and H-1 placing all the methyl groups (CH_3_-17, CH_3_-20, and CH_3_-18) on the same face of the molecule (Figure 4). The position of the hydroxyl group at C-9 was assigned to the opposite face of the methyl groups based upon strong NOE correlations observed between CH_3_-20 and H-11a. Furthermore, libertellenone M showed a similar relative configuration to those of the closely structurally related known compounds (**4**) and (**5**) supporting the assignment (Figure 2).

HRESITOFMS showed a 18.01 Da mass difference between compounds (**3**) ([M + H]^+^ *m*/*z* 345.1692, accuracy −0.76 ppm) and (**2**) suggesting the latter to be a dehydrated analogue of the former. Compound (**3**) degenerated gradually during the NMR run, complicating the interpretation of the NMR data and preventing further analysis and bioactivity studies of the compound. With this in regard, ^1^H, DQF-COSY and HSQC spectra of compounds (**3**) and (**2**) were highly similar, suggesting compound (**3**) to be the opened γ-lactone ring of libertellenone M (**2**) formed through hydrolysis of the ester. Further in the HMBC experiment, key correlations were observed resembling those exhibited by compound (**2**) and with the most notable difference in the carbon chemical shift seen at C-5 that shifted upfield to δ_C_ 137.0 ppm compared to 146.9 ppm for compound (**2**) (a similar upfield shift was observed for libertellenone C (**4**) in comparison to compound (**2**)). A table with NMR spectroscopic data is shown for both compounds (**2**) and (**3**) in Table 2. Analysis of the MS/HRMS data assisted in the confirmation of the structure of compound (**3**) to be the opened γ-lactone ring of libertellenone M (**2**). The major degeneration product of compound (**3**) was observed at *m*/*z* 299.1644 [M + H]^+^ (also present as a fragment ion in the MS/HRMS spectra of both compounds (**2**) and (**3**)) indicating the loss of HCO_2_H (∆ 46.0048 between fragment ion and [M + H]^+^, calcd. 46.0054). The three compounds displayed highly similar fragmentation patterns with similarity scores ≥84% (compound (**3**)) and ≥88% (*m*/*z* 299.1644) for all MS/HRMS spectra (10, 20 and 40 eV) compared to libertellenone M (**2**). These were observed searching the MS/HRMS spectra of both analogues against our MS/HRMS library spectra (libertellenone M included) by similarity scoring as in Kildgaard et al. [11], identifying compounds that share the same fragment ions but have different molecular masses. MS/HRMS spectra are shown for compounds (**2**) and (**3**) in Figures S2 and S3, Supplementary information. In addition compound (**2**) was observed as a minor degeneration product of compound (**3**), suggesting the relative configuration of both to be the same.

Myrocin F (**1**), libertellenone M (**2**), libertellenone C (**4**) and libertellenone E (**5**) were all evaluated for their activity towards GSCs and compounds (**1**), (**2**), and (**4**) were shown to display IC_50_ values of 40, 18, and 40 µM, respectively. The cytotoxicity of the diterpenoids was also evaluated towards the following cancer cell lines: A549 (lung carcinoma), MCF7 (breast carcinoma), SW480 (colorectal adenocarcinoma), and DU145 (prostate carcinoma). Myrocin F (**1**) showed the strongest effect with IC_50_ values between 20–50 µM, whereas compounds (**2**) and (**4**) proved to be much less cytotoxic towards these cell lines (See Figures S46 and S47 for data). Libertellenone E (**5**) did not display any activity towards any of the cell lines at the tested concentrations (IC_50_ > 300 µM). The higher cytotoxicity displayed against all cancer cell lines for myrocin F could indicate the cyclopropane ring’s influence on the bioactivity, in agreement with previous reports for other pimarane diterpenoids [38].

As far as antibacterial (*Escherichia* *coli* and methicillin-sensitive *S. aureus* (MSSA)) and antifungal activity (*Aspergillus fumigatus* and *Candida albicans*) was concerned, none of the compounds were active at the concentrations tested (MIC_90_ > 64 µg/mL).

### 2.2. Ilicicolin H, A Broad-Spectrum Antifungal, and New Analogues

**Figure 5:**
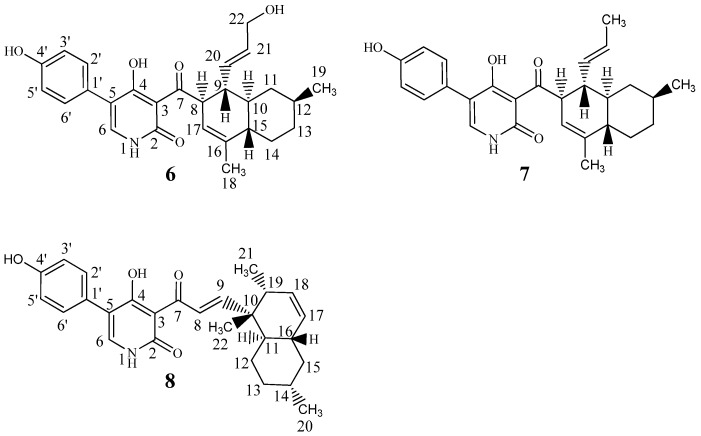
Structures of hydroxyl-ilicicolin H (**6**), ilicicolin H (**7**), ilicicolin I (**8**).

From the original small scale cultivation (eight plates) combined YES and CYA extract, the sixth flash fraction (ranging from 85% to 100% organic) displayed antifungal activity against *A*. *fumigatus*. UHPLC-DAD-QTOFMS revealed the molecular formula C_27_H_31_NO_4_ (0.7 ppm accuracy) for one of the major compounds in the fraction. AntiBase 2012 [41] suggested the broad spectrum antifungal compound, ilicicolin H (Figure 5, (**7**)) as a candidate, consistent with UV data [25] and the biological activity of the fraction [27,28]. Ilicicolin H is a hybrid polyketide—non-ribosomal peptide derived fungal metabolite that was originally isolated in 1971 from the ‘imperfect fungus’ *Cylindrocladium ilicicola* [42], with its structure elucidation described in 1976 [25], biosynthesis in 1983 [26], and total synthesis of racemic ilicicolin H in 1985 [43]. The production of this compound was highly increased when incubation time on rice media was extended from 10 days (as was optimal for the pimarane diterpenoids) to three weeks at 25 °C in the dark (Cultivation 3). This led to isolation of the compound in high amounts ( >50 mg) and 1D and 2D NMR confirmation (See Table 3) of the structure to be ilicicolin H, confirmed by comparing ^1^H- and ^13^C- chemical shifts to that of published data [25,26].

**Figure 6:**
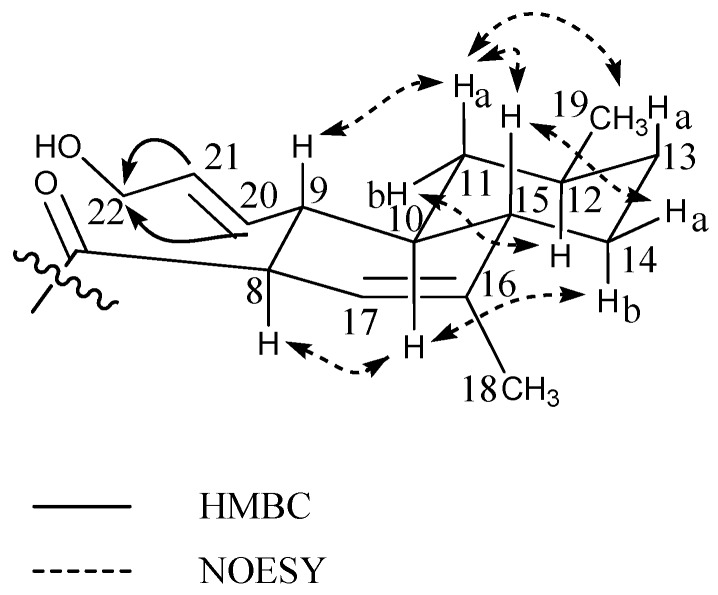
Selected important HMBC correlations (^1^H-^13^C) to C-22 and NOESY correlations for the decalin moiety of hydroxyl-ilicicolin H (**6**).

HRMS and MS/HRMS of a group of peaks eluting in close proximity to ilicicolin H in cultivation 3 showed the presence of several ilicicolin H analogues in searching their MS/HRMS spectra against our MS/HRMS library spectra (ilicicolin H included) by similarity scoring as in Kildgaard et al. [11]. Ilicicolin H and the tentatively identified analogues all shared the dominant fragment ion at *m*/*z* 230.0451 that corresponds to the left hand part of the molecule (Figure 5) with incorporation of phenylalanine, [C_12_H_8_NO_4_]^+^ formed from cleavage of the C-7/C-8 bond. See Figure S5, Supplementary information for BPC of the crude rice extract with EIC from MS/HRMS showing the fragment ion *m*/*z* 230.0451 and EIC from MS displaying ilicicolin H (434.2323 calculated for [C_27_H_31_NO_4_ + H]^+^) and the tentatively identified analogues and their position in the chromatogram. Two new analogues, hydroxyl-ilicicolin H (**6**) and ilicicolin I (**8**) (See Figure 5) were isolated from the crude rice extract and their structures elucidated by NMR spectroscopy (See Table 3). The first new analogue (**6**) was purified in low amounts (0.4 mg), eluting slightly earlier than ilicicolin H in the ESI^+^ chromatogram. The ESI^+^ HRMS spectrum displayed the pseudomolecular ion, [M + H]^+^ with *m*/*z* 450.2278, from which the molecular formula could be deduced as C_27_H_31_NO_5_ (accuracy −1.23 ppm), indicating the addition of an oxygen atom. In relation to ilicicolin H, a similarity score of 90% was observed by comparing the MS/HRMS spectra at 40 eV and the same absorptions maxima at 250 nm, 295 nm, and 350 nm were displayed in the UV spectra. The structure of hydroxyl-ilicicolin H (**6**) was proposed from 1D and 2D NMR spectroscopic analysis (See Table 3 for ^1^H and ^13^C chemical shifts). The NMR data for the phenyl-pyridone moiety were comparable to those of ilicicolin H elucidated in the same solvent. For the decalin moiety, the ^1^H-NMR spectrum exhibited eight methines (including three vinylic), four methylenes (including three diastereotopic), and two methyl groups (including one singlet). This indicated the difference of an oxidation of the methyl group at δ_H_ 1.53 (CH_3_-22) in ilicicolin H to the enantiotopic methylene at δ_H_ 3.85 (CH_2_-22) in hydroxyl-ilicicolin H, with the observed downfield chemical shift of the methylene supporting the presence of the hydroxyl group at C-22. The position of the CH_2_-22 was confirmed by identification of observed vicinal couplings in the COSY spectrum between δ_H_ 5.47 (CH-21) and δ_H_ 3.85 (CH_2_-22) belonging to the spin system including CH-8 to CH-15, CH-17, CH_3_-19 and CH-20 to CH_2_-22. Furthermore, HMBC correlations were observed from the vinylic protons at δ_H_ 5.41 (CH-20) and δ_H_ 5.47 (CH-21) to the methylene carbon at δ_C_ 63.5 (C-22) (See Figure 6). The relative configuration of hydroxyl-ilicicolin H (**6**) was suggested to be the same as ilicicolin H (**7**), based on the inspection of coupling constants and observed NOEs in the NOESY spectra (See Figure 6) together with the close structural similarity of compounds (**6**) and (**7**). A trans diaxial relationship was suggested for the protons at H-8 and H-9 and H-9 and H-10, as H-9 was observed as a quartet in the ^1^H-NMR spectrum with a coupling constant *J* = 10.4 Hz. Furthermore, the proton at δ_H_ 0.61 (H-11a) also appeared in the ^1^H-NMR spectrum as a quartet with a coupling constant *J* = 11.8 Hz, indicating a trans diaxial relationship between the protons H-10 and H-11a and H-11a and H-12. This was supported by observed NOEs of H-10 with H-8, H-11b and H-14b, and of H-11b with H-12 that placed these protons on the same face of the molecule, whereas NOEs of H-11a with H-9, H-15, CH_3_-19 and H-13a and of H-15 with H-9, H-13a and H-14a placed these protons on the opposite face of the molecule. The size of the vicinal coupling constants for H-20/H-21 of *J* = 15.5 Hz suggested trans stereochemistry and NOEs were observed of H-20 with H-11b and of H-21 with H-9.

**Figure 7:**
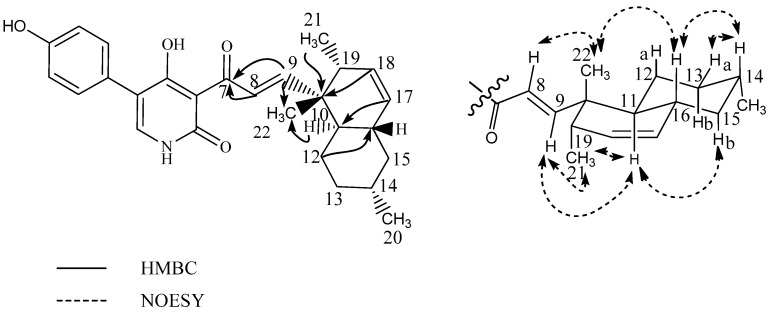
Selected important HMBC correlations (^1^H-^13^C) and NOESY correlations for the decalin moeity of ilicicolin I (**8**).

The second new analogue (**8**) was observed eluting as a small peak at almost the same retention time as ilicicolin H in the ESI^+^ chromatogram and it was deduced to possess the same molecular formula of C_27_H_31_NO_4_ ([M + H]^+^ with *m*/*z* 434.2325, −0.20 ppm accuracy), indicating the presence of an ilicicolin H isomer. The compound showed a similarity score of 92% (MS/HRMS spectra, 40 eV) and the UV spectrum displayed a slight bathochromic shift to longer wavelength with absorption maxima at 254 nm, 308 nm, and 365 nm to that of ilicicolin H. The structure of ilicicolin I (**8**) was elucidated by 1D and 2D NMR spectroscopic analysis (See Table 3). The NMR data for the phenyl-pyridone moiety were comparable to those of ilicicolin H, whereas observed changes to the decalin moiety revealed the compound to be a structural isomer. The ^1^H-NMR spectrum for the decalin moiety displayed eight methines (including four vinylic), three methylenes (all diastereotopic), and three methyl groups (including one singlet). The DQF-COSY spectrum defined two spin systems besides the singlet methyl group at δ_H_ 1.10 (CH_3_-22). One spin system consisted of the two vinylic protons at δ_H_ 7.98 (CH-8) and δ_H_ 7.26 (CH-9) with the size of the coupling constant of J_89_ = 16.0 Hz, indicating trans stereochemistry. The second spin system consisted of the two vinylic methines at δ_H_ 5.41 (CH-17) and δ_H_ 5.58 (CH-18), four methines at δ_H_ 1.41 (CH-11), δ_H_ 1.47 (CH-14), δ_H_ 1.81 (CH-16) and δ_H_ 1.91 (CH-19), three diastereotopic methylenes at δ_H_ 1.40/1.07 (CH_2_-12), δ_H_ 1.73/1.00 (CH_2_-13), and δ_H_ 1.80/0.80 (CH_2_-15) and two methyl groups at δ_H_ 0.90 (CH_3_-20) and δ_H_ 0.98 (CH_3_-21). The linking between these two COSY spin systems and the phenyl-pyridone moiety together with the assignment of the singlet methyl group at CH_3_-22 and quaternary carbons was accomplished by analysis of the HMBC spectrum (See Figure 7). Important HMBC correlations from the vinylic protons H-8 and H-9 to the ketone at δ_C_ 195.7 (C-7) assisted in the connection of this spin system to the phenyl-pyridone moiety. The upfield shift observed for the ketone at C-7 (decreasing from 211.0 to 195.7 ppm) in comparison to ilicicolin H and the downfield shift of the β carbon at δ_C_ 160.2 (C-9) supports that the C-8/C-9 double bond is in conjugation with the ketone at C-7. HMBC correlations from H-8 and H-9 to δ_C_ 42.6 (C-10), H-9 to δ_C_ 18.5 (C-22), H-12, H-18, H-21, and H-22 to C-10 and H-11 and H-19 to C-22 linked the spin system of CH-8 and CH-9 to the remaining spin system of the polyketide chain (including CH-11 to CH-21) via the quaternary carbon at C-10 and singlet methyl group at C-22. Further key HMBC correlations from H-12 and H-18 to δ_C_ 39.3 (C-16) and from H-15 and H-17 to δ_C_ 43.2 (C-11) assisted in the assembly of the decalin ring system. The relative stereochemistry of ilicicolin I was proposed based on coupling constants and observed NOEs in the NOESY experiments (See Figure 7). A trans diaxial relationship was suggested for the protons H-16 and H-15b and H-15b and H-14 based on the observation that H-15b appears as a quartet in the ^1^H-NMR spectrum with a coupling constant of *J* = 12.5 Hz. Furthermore, a trans diaxial relationship was also assumed for the protons H-14 and H-13b, H-13b, and H-12a and H-12a and H-11 based on H-13b and H-12a appearing in the ^1^H-NMR spectrum as double quartets with coupling constants of *J* = 12.4 and 3.4 Hz. This was supported by observed NOEs of H-8 with CH_3_-22, CH_3_-22 with H-16 and H-19, H-16 with H-14 and H-15a, H-14 with H-12a and H-13a placing these protons on the same side of the plane, whereas correlations of H-9 with CH_3_-21, H-11, and H-12b, H-11 with H-15b and CH_3_-21, and of H-12b with H-13b placed these protons on the opposite side of the plane.

Based on the structural similarities between ilicicolin I and ilicicolin H, we hypothesize that the decalin moiety for ilicicolin I is biosynthesized by the fungus via cyclization between C-10 and C-19 and between C-11 and C-16 through an intermolecular Diels-Alder reaction of the reduced octaketide chain instead of cyclization between C-8 and C-9 and C-10 and C-15 as in ilicicolin H [26].

Two other ilicicolin H analogues with the pseudomolecular ions, [M + H]^+^ *m*/*z* 452.2436 (C_27_H_33_NO_5_, accuracy −1.26 ppm) and *m*/*z* 420.2126 (C_26_H_29_NO_4_, accuracy −0.87 ppm) were tentatively identified by HRMS and MS/HRMS to also elute in close proximity to ilicicolin H. This suggested the new structures with the possible addition of H_2_O for the former and one less methyl group (difference of a CH_2_ unit) for the latter compound compared to ilicicolin H. The two compounds shared the dominant fragment ion at *m*/*z* 230.0451 and showed similarity scores of 80% and 92% to that of ilicicolin H for their 40 eV MS/HRMS spectra, respectively (See Figures S5 and S6, Supplementary information for MS/HRMS spectra). Only trace amounts insufficient for purification were present of these two possibly new analogues in the crude extract.

Hydroxyl-ilicicolin H (**6**) and ilicicolin I (**8**) were evaluated together with ilicicolin H (**7**) for their antifungal activity against *A. fumigatus*, *C. albicans, Candida parapsilosis,* and *Candida tropicalis*. Compounds (**6**) and (**8**) did not show any activity at the tested concentrations (MIC_90_ > 128 µg/mL). In contrast, ilicicolin H (**7**) exhibited strong activities against *A. fumigatus* (MIC_90_ 0.5–1 µg/mL), *C. albicans* (MIC_90_ < 0.25 µg/mL), and *C. parapsilosis* (MIC_90_ 0.5 µg/mL) to confirm the observed activity of the antifungal fraction. Structure–activity relationship (SAR) studies have previously been performed during structural modifications (chemical, biotransformation, and enzymatic) to ilicicolin H [27,28,44]. As shown here, a series of semisynthetic analogues produced by biotransformation of ilicicolin H generally showed a significant loss of activity when oxidized [44]. The importance of the β-diketone feature (C-4-C-3-C-7) has been indicated in the antifungal activity and in general a significant reduction or loss of activity has been seen for compounds with modification around the β-diketone and hindrance of an established bioactive conformation with a perpendicular orientation between the left hand phenyl-pyridone side and right hand decalin side [27,28]. The loss of activity seen for ilicicolin I may be due to the hindrance of this structural isomer to take up the right bioactive conformation.

## 3. Materials and Methods

### 3.1. General Experimental Procedures

UHPLC-DAD-QTOFMS was performed on an Agilent Infinity 1290 UHPLC system (Agilent Technologies, Santa Clara, CA, USA) equipped with a DAD. Separation was achieved on an Agilent Poroshell 120 phenyl-hexyl column (2.1 × 150 mm, 2.7 µm) with a flow rate of 0.35 mL/min at 60 °C using a linear gradient of 10% acetonitrile (MeCN) in Milli-Q water buffered with 20 mM formic acid (FA) increased to 100% in 15 min staying there for 2 min, returned to 10% in 0.1 min and kept there for 3 min before the following run. MeCN was LC-MS grade. MS detection was done on an Agilent 6545 QTOF MS equipped with Agilent Dual Jet Stream electrospray ion source with a drying gas temperature of 160 °C, a gas flow of 13 L/min, and a sheath gas temperature of 300 °C and flow of 16 L/min. Capillary voltage was set to 4000 V and a nozzle voltage to 500 V. Other MS parameters including description of the automated data-dependent MS/HRMS (at 10, 20, and 40 eV) can be found in Kildgaard et al. 2014 [11]. The MS data were analyzed in three different ways. First, full scan HRMS data were data mined (aggressive dereplication) for [M + H]^+^, [M + Na]^+^, [M − H]^−^, and [M + HCOO]^−^ adducts of all known elemental compositions described from *Stilbella* and related genera [11], here the mass accuracy, isotopic ratios, and isotopic spacing [45] were added into a combined score (0–100%), where only hits above 70% were considered. Secondly, the MS/HRMS spectra were searched against the in-house library using the Agilent MassHunter PCDL manager (Agilent Technologies), with 20 ppm accuracy on the parent ion and 30 ppm on the fragment ions, giving a score of 0–100%. Finally, the elemental composition of peaks not identified in the previous two steps, were identified based on the mass accuracy, isotopic ratios, and isotopic spacing (sometimes providing several candidates above 70%). Then similarity search (>50% of 100%) was used for matching peaks in the library spectrum against the unknown spectrum (independent on parent mass) [11] to pinpoint related pimarane diterpenoids and hybrid polyketide-non ribosomal peptides, since both had groups displaying unique and very different fragment ions (Supplementary information). Pre-fractionation was performed using flash chromatography of the crude extract with an Isolera One automated flash system (Biotage, Uppsala, Sweden). Purification of compounds was conducted using a Waters 600 Controller (Milford, MA, USA) coupled to a Waters 996 Photodiode Array Detector. One and two dimensional NMR experiments were acquired using standard pulse sequences on a 400 MHz Bruker Ascend spectrometer with a Prodigy cryoprobe, 600 MHz Bruker Ascend with a SmartProbe (BBO) and a 800 MHz Bruker Avance spectrometer with a 5 mm TCI cryoprobe, alternatively on a 500 MHz Bruker Avance with a 1.7 mm cryoprobe at Fundación Medina, Spain. Optical rotations were measured on a Perkin Elmer 341 polarimeter (Perkin Elmer, Waltham, MA, USA).

### 3.2. Fungal Strain and Identification

The filamentous fungus was isolated from a sea water sample off the coast of the Danish island Fanoe. The fungus was 3-point inoculated on CYA, OAT, PDA, and V8 agar plates [46] and incubated at 25 °C in the dark. After 11 days of growth on V8, microscope slides were made and a morphological examination identified the fungus as *Stilbella fimetaria* (Pers.) Lindau. Molecular sequencing of the ITS region confirmed the morphological identification. The fungus (IBT 28361) is stored in the IBT culture collection at DTU Bioengineering, Technical University of Denmark.

### 3.3. Cultivation

Original small scale cultivation: the marine-derived fungus was 3-point inoculated on ten agar plates (five CYA and five YES) and incubated for 9 days in the dark at 25 °C [46]. Cultivation 1: the fungus was 3-point inoculated on 200 YES plates and incubated for 9 days in the dark at 25 °C. Cultivation 2: the fungus was inoculated into 6 × 1.8 L conical culture flasks with organic grain rice (150 g per flask) and Milli-Q water (150 g per flask) and incubated at 25 °C in the dark for 10 days. Cultivation 3: the fungus was inoculated into 15 small conical flasks 0.5 L with organic grain rice (50 g per flask) and Milli-Q water (50 g per flask) and incubated for 21 days at 25°C in the dark.

### 3.4. Extraction and Isolation

Original small scale cultivation: Extraction of the eight plates (four CYA and four YES) was achieved with 150 mL EtOAc containing 1% FA. The crude extract was then fractionated on a RP C_18_ flash column (Sepra ZT, Isolute, 10 g) using the Isolera One automated flash system. The gradient used was MeCN and water buffered with 20 mM FA going from 15% to 100% MeCN over 28 min (12 mL/min). Six flash fractions were automatically collected based on UV signal (210 nm and 254 nm). MeCN was of HPLC grade and water was purified and deionized by a Millipore system with a 0.22 µm membrane filter (Milli-Q water). For one CYA plate and one YES plate 4 plugs were taken from one colony with a 6-mm plug drill, covering the diameter of the colony and extracted with 1 mL EtOAc containing 1% FA and otherwise prepared in accordance with the micro-scale extraction method described by Smedsgaard [47].

Cultivation 1: Extraction was achieved with 150 mL EtOAc with 1% FA for every 10 plates. Liquid-liquid extraction was performed with 1:9 Milli-Q water:methanol (MeOH) and 1:1 heptane, resulting in two phases, the Milli-Q water/MeOH phase was added Milli-Q water to a ratio 1:1, and metabolites were extracted with dichloromethane (DCM). This was done to remove unwanted carbohydrates from the media as well as fatty acids. The crude extract from the DCM phase was fractionated on a RP C_18_ flash column (Sepra ZT, Isolute, 25 g) using the Isolera One automated flash system. The gradient used was 15–100% MeCN buffered with 20 mM FA over 28 min (25 mL/min). Fractions were automatically collected based on UV signal (210 nm and 254 nm). The bioactive fraction (going from 40–50% MeCN) was further fractionated on the Isolera system using a diol flash column (Diol, 25 g, 33 mL) and fractions were eluted with two column volumes (2 col. vols.) per fraction with DCM, DCM/EtOAc, EtOAc, EtOAc/MeOH, and MeOH with a flow rate of 25 mL/min. The bioactive fractions going from 50% to 60% and 60% to 85% MeCN were fractionated further on the Isolera system using a RP C_18_ flash column (10 g/15 mL). The gradient was 5% stepwise (13 col. vols.) from 35% to 100% MeOH buffered with 20 mM FA with a flow rate of 15 mL/min. Fractions were collected manually for every 5%. Myrocin F and helvolic acid were purified on the Waters 600 semi-preparative HPLC. Myrocin F separation was achieved on a Luna II C_18_, 5 μm, 250 × 10 mm column (Phenomenex, Torrance, CA, USA) with a flow rate of 5 mL/min using a linear gradient of 45% MeCN in Milli-Q water with 20 mM FA going to 75% MeCN in 20 min. Helvolic acid separation was achieved on a Luna II C_18_, 5 μm, 250 × 10 mm column (Phenomenex, Torrance, CA, USA) with a flow rate of 4 mL/min using a linear gradient 60% MeCN in Milli-Q water going to 100% MeCN in 20 min.

Cultivation 2: Extraction was achieved using 600 mL per flask of EtOAc with 1% FA. Liquid-liquid extraction was performed with 1:9 Milli-Q water:MeOH and 1:1 heptane, the Milli-Q water/MeOH phase was added Milli-Q water to a ratio 1:1, and metabolites were extracted with DCM, leaving the crude extract from the DCM phase. The crude extract was fractionated on a diol flash column (Diol, 25 g, 33 mL) and compounds were eluted with 2 col. vols. per fraction: heptane, heptane/DCM, DCM, DCM 3: 1 EtOAc, DCM/EtOAc, EtOAc, EtOAc 3:1 MeOH, EtOAc/MeOH, and MeOH. Fractions DCM 3: 1 EtOAc and DCM/EtOAc were further fractionated on a RP C_18_ column (15 μm/100 Å, 10 g/15 mL) using the Isolera One automated flash system. The gradient was 5% stepwise (13 col. vols.) from 35% to 100% MeOH buffered with 20 mM FA with a flow rate of 15 mL/min. Fractions were collected manually for every 5%. Libertellenone M, the suggested opened γ-lactone of libertellenone M and libertellenone C were purified on the Waters 600 semi-preparative HPLC. Libertellenone M and the opened γ-lactone of libertellenone M separation was achieved on a Gemini C_6_-Phenyl, 5 μm, 250 × 10 mm column (Phenomenex, Torrance, CA, USA) with a flow rate of 4 mL/min using a linear gradient 40% MeCN in Milli-Q water with 20 mM FA going to 100% MeCN in 28 min. Further libertellenone M separation was done on a Luna II C_18_, 5 μm, 250 × 10 mm column (Phenomenex, Torrance, CA, USA) with a flow rate of 4 mL/min isocratic 55% MeCN in Milli-Q water with 20 mM FA in 20 min and a Kinetex Biphenyl, 5 μm 250 × 10 mm column (Phenomenex, Torrance, CA, USA) with a flow rate of 4 mL/min using a linear gradient 30% MeCN in Milli-Q water with 20 mM FA going to 100% MeCN in 25 min. Libertellenone C separation was achieved on a Luna II C_18_, 5 μm, 250 × 10 mm column (Phenomenex, Torrance, CA, USA) with a flow rate of 5 mL/min using a linear gradient of 30% MeCN in Milli-Q water with 20 mM FA going to 70% MeCN in 30 min. Libertellenone E was purified from the EtOAc 3:1 MeOH fraction on the Waters 600 semipreparative HPLC. Separation was achieved on a Luna II C_18_, 5 μm, 250 × 10 mm column (Phenomenex, Torrance, CA, USA) with a flow rate of 5 mL/min using a linear gradient of 30% MeCN in Milli-Q water with 20 mM FA going to 70% MeCN in 20 min and a Kinetex Biphenyl, 5 μm 250 × 10 mm column (Phenomenex, Torrance, CA, USA ) with a flow rate of 4 mL/min using a linear gradient of 30% MeCN in Milli-Q water with 20 mM FA going to 100% MeCN in 25 min.

Cultivation 3: Extraction was achieved using 150 mL EtOAc per flask. Liquid-liquid extraction was performed with 1:9 Milli-Q water:MeOH and 1:1 heptane, the Milli-Q water/MeOH phase was added Milli-Q water to a ratio 1:1, and metabolites were extracted with DCM, leaving the crude extract from the DCM phase. The crude extract was pre-fractionated on a diol flash column (Diol, 25 g, 33 mL) and compounds were eluted with 2 col. vols. per fraction: heptane, heptane/DCM, DCM, DCM 3:1 EtOAc, DCM/EtOAc, EtOAc, EtOAc 3:1 MeOH, EtOAc/MeOH, and MeOH. Interesting fractions were further fractionated on a RP C_18_ column (15 μm/100 Å, 10 g/15 mL) using the Isolera One automated flash system. The gradient was 5% stepwise (13 col. vols.) from 35% to 100% MeOH buffered with 20 mM FA with a flow rate of 15 mL/min. Fractions were collected manually for every 5%. Ilicicolin H and hydroxy-ilicicolin H purification was achieved from the 80% MeOH and 50% MeOH fractions, respectively, on a Gemini C_6_-Phenyl, 5 μm, 250 × 10 mm column (Phenomenex, Torrance, CA, USA) with a flow rate of 4 mL/min using a linear gradient from 80% MeCN in Milli-Q water with 20 mM FA going to 100% MeCN in 15 min for ilicicolin H and from 50% MeCN in Milli-Q water with 20 mM FA going to 90% MeCN in 20 min for hydroxy-ilicicolin H. Ilicicolin I was purified from the 60% MeOH fraction on a Kinetex Biphenyl, 5 μm 250 × 10 mm column (Phenomenex, Torrance, CA, USA ) with a flow rate of 4 mL/min using an isocratic gradient at 75% MeCN in Milli-Q water with 20 mM FA for 20 min.

**Helvolic acid**: white solid; UV (MeCN) λ_max_: 234 nm; ^13^C NMR see Figure S12 and Table 1; HRESIMS *m*/*z* 591.2932 ([M + Na]^+^ calculated for C_33_H_44_O_8_Na, *m*/*z* 591.2922)
**Myrocin F**: white solid; UV (MeCN) λ_max_: 215 nm, 270 nm; ^13^C- and ^1^H-NMR see Table 1; HRESIMS *m*/*z* 329.1745 ([M + H]^+^ calculated for C_20_H_25_O_4_, *m*/*z* 329.1746)
**Libertellenone M**: white solid; ${\left[\alpha \right]}_{D}^{20}$ −81° (*c* 0.10, MeOH); UV (MeCN) λ_max_: 220 sh nm, 270 sh nm, 290 nm; ^13^C- and ^1^H-NMR see Table 2; HRESIMS *m*/*z* 327.1592 ([M + H]^+^ calculated for C_20_H_23_O_4_, *m*/*z* 327.1590)
**Opened γ-lactone ring of libertellenone M**: white solid; UV (MeCN) λ_max_: 220 sh nm, 270 nm, 315 nm; ^13^C- and ^1^H-NMR see Table 2; HRESIMS *m*/*z* 345.1692 ([M + H]^+^ calculated for C_20_H_25_O_5_, *m*/*z* 345.1695)
**Libertellenone C**: white solid; ${\left[\alpha \right]}_{D}^{20}$ −98° (*c* 0.11, MeOH); UV (MeCN) λ_max_: 218 nm, 270 nm, 325 nm; ^13^C- and ^1^H-NMR see Figure S31 and Table 2; HRESIMS *m*/*z* 349.2012 ([M + H]^+^ calculated for C_20_H_29_O_5_, *m*/*z* 349.2007)
**Libertellenone E**: white solid; ${\left[\alpha \right]}_{D}^{20}$ +24.6° (*c* 0.13, MeOH); UV (MeCN) λ_max_: 214 nm, 268 nm, 314 nm; ^13^C- and ^1^H-NMR see Figure S31 and Table 2; HRESIMS *m*/*z* 347.1858 ([M + H]^+^ calculated for C_20_H_27_O_5_, *m*/*z* 347.1851)
**Ilicicolin H**: yellow solid; ${\left[\alpha \right]}_{D}^{20}$ −159° (*c* 0.11, MeOH); UV (MeCN) λ_max_: 250 nm, 295 nm, 350 nm; ^13^C- and ^1^H-NMR see Table 3; HRESIMS *m*/*z* 434.2325 ([M + H]^+^ calculated for C_27_H_32_NO_4_, *m*/*z* 434.2323)
**Hydroxyl-ilicicolin H**: yellow solid; UV (MeCN) λ_max_: 250 nm, 295 nm, 350 nm; ^13^C- and ^1^H-NMR see Table 3; HRESIMS *m*/*z* 450.2278 ([M + H]^+^ calculated for C_27_H_32_NO_5_, *m*/*z* 450.2272)
**Ilicicolin I**: yellow solid; UV (MeCN) λ_max_: 254 nm, 308 nm, 365 nm; ^13^C- and ^1^H-NMR see Table 3; HRESIMS *m*/*z* 434.2325 ([M + H]^+^ calculated for C_27_H_32_NO_4_, *m*/*z* 434.2323

### 3.5. Cytotoxicity Assay

NCH421k GSCs were derived from primary GBM patients who underwent surgical resection according to the research proposals approved by the Institutional Review Board at the Medical Faculty of Heidelberg. Tissues were enzymatically dissociated and cells were cultivated as floating neurospheres under standard conditions (37 °C, 95% humidity, and 5% CO_2_) in serum-free stem cell medium (DMEM/F-12 medium, 20% (*v*/*v*) BIT-admixture and 20 ng/mL each of basal fibroblast growth factor (bFGF) and epidermal growth factor (EGF)). Cells were generally cultivated in 75 cm^2^ untreated cell culture flasks (Sarstedt, Newton, MA, USA). When spheres reached around 150–300 µm in diameter, cells were passaged into new medium. Spheres were separated from debris and dead cells by gravity sedimentation, before suspension in 1 mL accutase and shaking at 1100 rpm at 37 °C. Accutase was removed after centrifugation at 900 g for 4 min and cells resuspended in 1 mL stem cell medium. Cells were passaged at 1:5–1:10 into 13 mL fresh stem cell medium, depending on the density of the previous culture. Malignant cell lines A549 (lung carcinoma), MCF7 (breast adenocarcinoma), SW480 (colorectal adenocarcinoma) and DU 145 (prostate carcinoma) were cultivated adherently in DMEM supplemented with 10% FCS and 1% (*v*/*v*) penicillin/streptomycin. *Stilbella fimetaria* extracts were initially tested for anticancer activity in NCH421k cells. To this end, cells were seeded in 96-well plates (Greiner, Munich, Germany) at a density of 20,000 cells per well in 100 µL medium. Dried fractionated fungal extracts were dissolved in DMSO and 10, 2, 0.4, 0.1, and 0.025 µg/well was applied to the cells. 48 h after incubation under standard cell culture conditions, cell viability was assessed using the CellTiter-Glo^®^ luminescent cell viability assay (Promega, Madison, WI, USA). Cells incubated with DMSO only were used as a control. In order to determine IC_50_ values for the pure diterpenoids, cells were seeded in 96-well plates (Greiner, Munich, Germany) at a density of 5000 cells per well for adherent cells and 8500 cells per well for NCH421k cells. Adherent cells were seeded 24 h in advance to allow the cells to attach. Compound was dissolved to 30 mM in 100% DMSO and three-fold serial dilutions were performed in cell culture medium. The compound containing medium was then applied with a dilution factor of ten, contributing to eight concentrations, starting at 300 µM for all the assays. Cell viability was assessed by the CellTiter-Glo^®^ (Promega, Madison, WI, USA) luminescent cell viability assay after 48 h incubation with the compound. Data were normalised to the DMSO control. Viability curves were plotted using Excel and IC_50_ values estimated from the curves. The assay was performed in biological triplicate.

### 3.6. Antibacterial and Antifungal Assays

Previously described methods were used for evaluating antibacterial and antifungal properties of extracts/compounds [48,49]. The pimarane diterpenoids were tested for their ability to inhibit the growth of Gram-negative and Gram-positive bacteria (*E. coli* ATCC25922 and MSSA MB2865), fungi (*A. fumigatus* ATCC46645) and yeast (*C. albicans* ATCC64124). Ilicicolin H and analogues were tested for their ability to inhibit the growth of *A. fumigatus* ATCC46645 and yeast (*C. albicans* ATCC64124, *C. parapsilosis* ATCC22019, and *C. tropicalis* ATCC750). Helvolic acid was tested for its ability to inhibit the growth of MRSA MB5393. Briefly, each compound was 3-fold serially diluted in DMSO with a dilution factor of 2 to provide 10 concentrations starting at 128 μg/mL for all the assays (for the pimarane diterpenoids only nine concentrations were used starting at 64 µg/mL). The MIC was defined as the lowest concentration of an antibacterial or antifungal compound that inhibited ≥90% of the growth of a microorganism after overnight incubation. The Genedata Screener software (Genedata, Inc., Basel, Switzerland) was used to process and analyse the data and also to calculate the RZ’ factor which predicts the robustness of an assay [50]. In all experiments performed in this work the RZ’ factor obtained was between 0.87 and 0.98.

## 4. Conclusions

In this study, our combined bio-guided and dereplication-based discovery approach of cytotoxicity and antimicrobial assays, UHPLC-DAD-QTOFMS-MS/HRMS using an in-house MS/HRMS library and pre-bioassay fractionation of a marine-derived fungus *Stilbella fimetaria* proved to be quick and effective in the identification of new and known bioactive natural products. There was no observed bioactivity for the *Stilbella fimetaria* crude extract on its own, whereas pre-fractionation allowed the observation of cytotoxicity, and antibacterial and antifungal activity, respectively, in three different fractions. This led to the discovery of several cytotoxic pimarane-type diterpenoids, including the two new diterpenes, myrocin F and libertellenone M, with IC_50_ values of 40 an 18 µM, respectively, towards patient derived glioblastoma stem-like cells. Myrocin F exhibited general cytotoxicity towards various cancer cell lines with IC_50_ values between 20 to 50 µM. The known broad-spectrum antifungal compound, ilicicolin H was revealed as the active compound contributing to the observed antifungal activity and MS/HRMS was applied to tentatively identify several new ilicicolin H analogues, including the two purified compounds, hydroxyl-ilicicolin H and ilicicolin I. Optimization on rice media allowed for the purification of compounds in the required amount for structure elucidation and bioassay analysis, with the production being optimal at around one week for the pimarane-type ditepenoids and three weeks for the ilicicolin H analogues.

## Figures and Tables

**Table 1:** NMR spectroscopic data (400 MHz, MeOD, δ in ppm, *J* in Hz) for myrocin F (**1**).

Position	δ^13^_C_	δ^1^_H_ (Mult, *J*)	HMBC	NOESY
1	14.0	1.63 m	3	2b,5,11,20b
2a	19.8	1.78 m	3,4	3a,20a
2b		1.81 m	3,4	1,5,18
3a	28.8	1.44 m	1,4,5,19	2a,20a
3b		1.74 m	4,19	18
4	42.8	-		
5	52.5	2.11 s	4,6,9,10,18–20	1,2b,7,16a,18
6	107.6	-		
7	77.4	4.24 s	5,6,8,9,14	5,14
8	135.5	-		
9	138.6	-		
10	20.5	-		
11	114.6	5.24 t(4.5)	8,10,12–14	1,12,20b
12	37.0	2.19 m	9,11,13–15,17	11,15,17
13	38.9	-		
14	135.8	5.56 s	7,9,11–13,15,17	7,16a,17
15	144.0	5.67 dd(17.4/10.4)	13,14,17	12,16a/b,17
16a	112.3	5.03 dd (17.4/1.5)	13	5,14,15,17
16b		4.89 dd(10.4/1.5)	13	15
17	28.0	1.15 s	12–15	12,14,15,16a
18	29.6	1.42 s	3–5,19	2b,3b,5
19	185.6	-		
20a	17.1	0.85 t(5.2)	1,5,9	2a,3a
20b		0.25 dd(8.2/5.7)	2,9	1,11

**Table 2:** NMR spectroscopic data (800 MHz CD_3_CN, δ in ppm, *J* in Hz) for libertellenone M (**2**) and (800 MHz MeOD, δ in ppm, *J* in Hz) for the suggested opened γ-lactam libertellenone M (**3**).

	Libertellenone M (2)			Opened γ-lactam libertellenone M (3)		
Position	δ^13^_C_	δ^1^_H_ (Mult, *J*)	HMBC	δ^13^_C_	δ^1^_H_ (Mult, *J*)	HMBC
1	130.7	5.78 dd(9.9,3.0)	3,5,6,10,20	130.7	5.94 m	3,10
2	127.4	5.91 m	3,4,10	126.7	5.98 m	3,10
3a	34.3	2.36 dt(16.5,2.5)	1,2,4,5,18	36.2	2.16 m	-
3b		2.43 dd(16.5,5.9)	1,2,4,18,19		2.64 m	1,2
4	46.2	-		46.8	-	-
5	146.9	-		137.0	-	-
6	143.1	-		*	-	-
7	177.3	-		183.6	-	-
8	137.6	-		*	-	-
9	76.6	-		76.0	-	-
10	45.5	-		46.6	-	-
11a	27.5	2.24 m	9,10,12,13	27.4	2.16 m	-
11b		1.72 ddd(14.0,5.0,3.5)	8–10,12,13		1.93 m	-
12a	30.9	1.59 m	9,11,14,17	30.6	1.60 m	-
12b		1.78 td(13.0,3.5)	9,11,13–15,17		1.92 m	17
13	39.8	-		40.0	-	-
14	148.8	6.90 s	7–9,12,13,15,17	148.8	6.98 s	7,9,12,15
15	147.0	5.93 m	12–14,17	147.0	5.92 m	-
16a	113.5	5.09 d(17.5)	13,15	113.0	5.12 d(17.2)	13
16b		5.07 d(10.5)	13,15		5.06 d(10.5)	13
17	24.8	1.17 s	12–15	23.8	1.16 s	12–15
18	23.4	1.48 s	3–5,19	24.1	1.55 s	3–5,19
19	181.2	-		181.1	-	
20	24.1	1.29 s	1,5,9,10	28.3	1.23 s	1,5,9,10

* ^13^C chemical shift not observed.

**Table 3:** NMR spectroscopic data (500 MHz and 800 MHz, MeCN-d3, δ in ppm, *J* in Hz) for hydroxyl-ilicicolin H (**6**), ilicicolin H (**7**), and ilicicolin I (**8**).

	Hydroxyl-ilicicolin H (6)		Ilicicolin H (7)		Ilicicolin I (8)	
Position	δ^13^_C_	δ^1^_H_ (Mult, *J*)	δ^13^_C_	δ^1^_H_ (Mult, *J*)	δ^13^_C_	δ^1^_H_ (Mult, *J*)
1′	125.9	-	125.8	-	126.0	-
3′5′	116.4	6.83 d(8.6)	116.6	6.83 d(8.6)	116.4	6.84 d(8.6)
2′6′	131.8	7.27 d(8.6)	131.7	7.26 d(8.6)	131.8	7.29 d(8.6)
4′	157.8	-	157.8	-	157.9	-
4′OH	-	16.7 br.s.	-	17.6 br.s.	-	-
1NH	-	9.46 br.s.	-	9.56 br.s.	-	9.44 br.s.
2	163.0	-	162.9	-	163.3	-
3	108.7^*^	-	108.1	-	107.5	-
4	178.2	-	178.0	-	179.5	-
5	114.9	-	114.8	-	115.1	-
6	141.3	7.40 s	141.4	7.40 s	141.2	7.42 s
7	210.8	-	211.0	-	195.7	-
8	54.1	4.98 m	54.1	4.97 m	127.5	7.98 d(16.0)
9	45.7	2.56 q(10.4)	46.2	2.48 q(10.4)	160.2	7.26 d(16.0)
10	44.5	1.28 m	44.5	1.23 m	42.6	-
11a	40.6	0.61 q(11.8)	40.6	0.58 q(11.8)	43.2	1.41 m
11b		1.78 m		1.77 m		
12a	33.8	1.40 m	33.8	1.38 m	28.5	1.07 dq(12.4,3.4)
12b						1.40 m
13a	36.6	0.97 m	36.6	0.97 m	36.8	1.73 m
13b		1.76 m		1.77 m		1.00 dq(12.5,3.4)
14a	31.0	2.07 m	31.0	2.04 m	34.3	1.47 m
14b		0.99 m		0.99 m		
15a	45.6	1.70 m	45.4	1.68 m	43.1	1.80 m
15b						0.80 q(12.5)
16	139.5	-	139.5	-	39.3	1.81 m
17	121.0	5.22 s	120.9	5.21 m	131.1	5.41 d(10.0)
18	21.4	1.65 s	21.5	1.63 s	132.5	5.58 ddd(10.0,4.7,2.6)
19	23.3	0.90 d(6.5)	23.4	0.89 d(6.5)	44.5	1.91 m
20	134.1	5.41 dd(15.5,8.2)	134.8	5.21 m	23.2	0.90 d(6.5)
21	132.6	5.47 dt(15.5,5.1)	127.3	5.32 m	18.6	0.98 d(7.0)
22	63.5	3.85 d(4.8)	18.5	1.53 d(6.5)	18.5	1.10 s

* very weak carbon chemical shift.

## Supplementary Materials

The following are available online at www.mdpi.com/1660-3397/15/8/253/s1, HRESITOFMS, MS/HRMS, UV and 1D and 2D NMR data of all new compounds are provided.
