# Supplementary material for: A Dereplication and Bioguided Discovery Approach to Reveal New Compounds from a Marine-Derived Fungus Stilbella fimetaria
Source: Mar Drugs. 2017 Aug 13;15(8):253. doi: 10.3390/md15080253 (PMC5577607; doi:10.3390/md15080253)

**Supporting Information**

**for**

**A dereplication and bioguided discovery approach to reveal new compounds from a marine-derived fungus *Stilbella fimetaria***

Sara Kildgaard, Karolina Subko, Emma Phillips, Violaine Goidts, Mercedes de la Cruz, Caridad Díaz, Charlotte Held Gotfredsen, Birgitte Andersen, Jens C. Frisvad, Kristian F. Nielsen and Thomas O. Larsen

Page S4. Figure S1. MS and UV and MS/HRMS (20 eV) spectra of myrocin F

Page S5. Figure S2. MS, MS/HRMS (10, 20 and 40 eV) and UV spectra of libertellenone M

Page S6. Figure S3. MS, MS/HRMS (10, 20 and 40 eV) and UV spectra of opened γ-lactam

libertellenone M

Page S7. Figure S4. BPC of the rice crude extract with EIC from HRMS showing the most abundant ion of the diterpenes: libertellenone E (*m/z* 369.1678 $[M+Na]^+$), libertellenone C (*m/z* 331.1907 $[M+H-H_2O]^+$), opened γ-lactone ring of libertellenone M libertellenone M (*m/z* 367.1512 $[M+Na]^+$) and libertellenone M (*m/z* 349.1404 $[M+Na]^+$)

Page S7. Figure S5. BPC of the rice crude extract with EIC from MS/HRMS showing the fragment ion *m/z* 230.0451 and EIC from MS of *m/z* 250.2278, *m/z* 452.2436, *m/z* 420.2126 and *m/z* 434.2325 displaying ilicicolin H and the tentatively identified analogues and their position in the chromatogram

Page S8. Figure S6. MS/HRMS spectra, 40 eV of *m/z* 250.2278, *m/z* 452.2436, *m/z* 420.2126 and *m/z* 434.2325 compared to the library spectrum of ilicicolin H. UV spectrum of ilicicolin H.

Page S8. Figure S7 MS and UV spectra of hydroxyl ilicicolin H

Page S9. Figure S8 MS and UV spectra of ilicicolin I

Page S10. Figure S9 $^1H$ NMR (600 MHz $CDCl_3$) of helvolic acid

Page S10. Figure S10 $^{13}C$ NMR (600 MHz $CDCl_3$ ) of helvolic acid

Page S11. Figure S11 Table 1 $^{13}$ C-NMR Spectroscopic Data (600 MHz $CD_3Cl$, δ in ppm) for helvolic acid

Page S12. Figure S12 $^1H$-NMR 400 MHz MeOD of myrocin F

Page S12. Figure S13. $^{13}C$-NMR 400 MHz MeOD of myrocin F

Page S13. Figure S14. COSY spectrum (400 MHz MeOD) of myrocin F

Page S13. Figure S15. Edited HSQC spectrum (400 MHz MeOD) of myrocin F

Page S14. Figure S16. HMBC spectrum (400 MHz MeOD) of myrocin F

Page S14. Figure S17. NOESY spectrum (400 MHz MeOD) of myrocin F

Page S15. Figure S18. $^1$H NMR (800 MHz $CD_3CN$) of libertellenone M

Page S15. Figure S19. $^{13}$C NMR (800 MHz $CD_3CN$) of libertellenone M

Page S16. Figure S20. DQF-COSY spectrum ( 800 MHz $CD_3CN$) of libertellenone M

Page S16. Figure S21. Edited HSQC (800 MHz $CD_3CN$) of libertellenone M

Page S17. Figure S22. HMBC spectrum (800 MHz $CD_3CN$) of libertellenone M

Page S17. Figure S23. NOESY spectrum (800 MHz $CD_3CN$) of libertellenone M

Page S18. Figure S24. $^1$H-NMR (800 MHz MeOD) of opened $\gamma$-lactam libertellenone M

Page S18. Figure S25. DQF-COSY spectrum (800 MHz $CD_3CN$) of opened $\gamma$-lactam libertellenone M

Page S19. Figure S26. Edited HSQC (800 MHz $CD_3CN$) of opened $\gamma$-lactam libertellenone M

Page S19. Figure S27. HMBC spectrum (800 MHz $CD_3CN$) of opened $\gamma$-lactam libertellenone M

Page S20. Figure S28. $^1$H-NMR (800 MHz $CD_3CN$) of libertellenone C

Page S20. Figure S29. $^{13}$C-NMR (800 MHz $CD_3CN$) of libertellenone C

Page S21. Figure S30. $^1$H-NMR (800 MHz $CD_3CN$) of libertellenone E

Page S22. Figure S31: Table 2. NMR Spectroscopic Data (400 MHz, MeCN-d3, $\delta$ in ppm) for libertellenone C and libertellenone E.

Page S23. Figure S32. $^1$H-NMR (500 MHz $CD_3CN$) of hydroxyl-ilicicolin H

Page S23. Figure S33. $^{13}$C-NMR (500 MHz $CD_3CN$) of hydroxyl-ilicicolin H

Page S24. Figure S34. COSY spectrum (500 MHz $CD_3CN$) of hydroxyl-ilicicolin H

Page S24. Figure S35. Edited HSQC (500 MHz $CD_3CN$) of hydroxyl-ilicicolin H

Page S25. Figure S36. HMBC spectrum (500 MHz $CD_3CN$) of hydroxyl-ilicicolin H

Page S25. Figure S37. NOESY spectrum (500 MHz $CD_3CN$) of hydroxyl-ilicicolin H

Page S26. Figure S38. $^1$H-NMR (800 MHz $CD_3CN$) of ilicicolin I

Page S26. Figure S39. $^{13}$C-NMR (800 MHz $CD_3CN$) of ilicicolin I

Page S27. Figure S40. COSY spectrum (800 MHz $CD_3CN$) of ilicicolin I

Page S27. Figure S41. HSQC spectrum (800 MHz $CD_3CN$) of ilicicolin I

Page S28. Figure S42. HMBC spectrum (800 MHz $CD_3CN$) of ilicicolin I

Page S28. Figure S43. NOESY spectrum (800 MHz $CD_3CN$) of ilicicolin I

Page S29. Figure S44. $^{1}$H-NMR (800 MHz $CD_3CN$) of ilicicolin H

Page S29. Figure S45. $^{13}$C-NMR (800 MHz $CD_3CN$) of ilicicolin H

Page S30. S46: Table 3. Anticancer activity of pimarane-diterpenes. $IC_{50}$ values (μM) of myrocin F, libertellenone M, libertellenone C and libertellenone E against cell lines NCH421k , A549, MCF7, SW480, DU 145 after incubation of each compound at (0 – 300 μM) for 48 hours.

Page S30. S47: Dose response curves of diterpenes in glioblastoma stem-like cells.

S1: MS and UV and MS/HRMS (20 eV) spectra of myrocin F

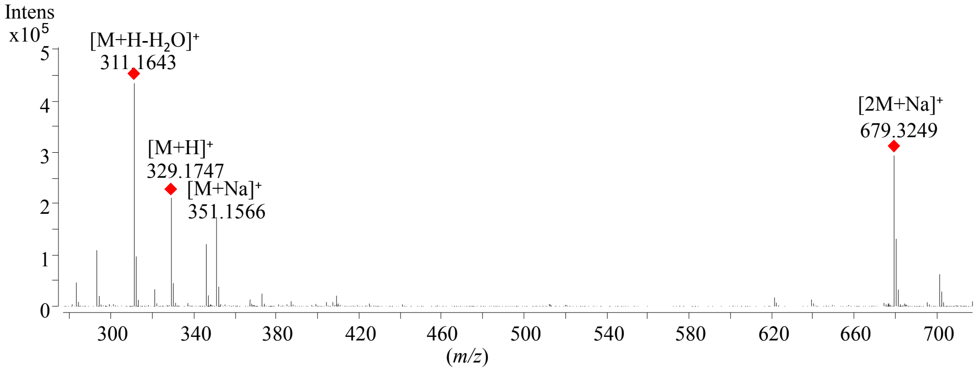


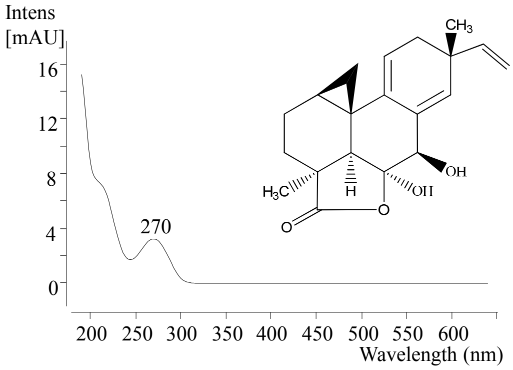


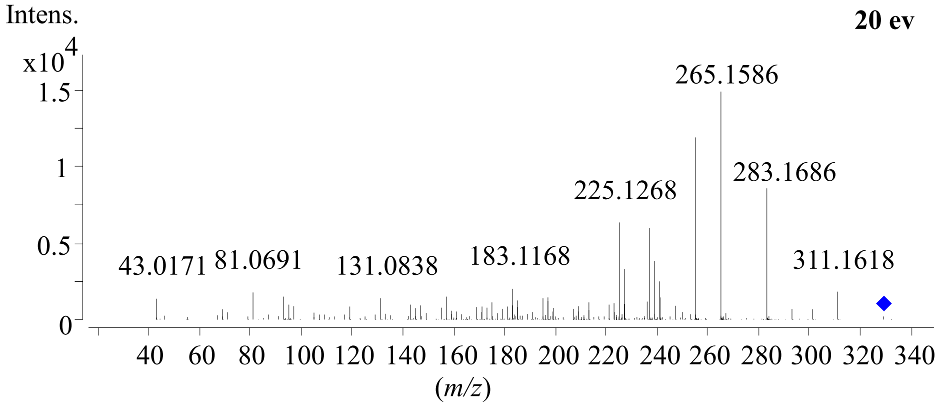

S2: MS, MS/HRMS (10, 20 and 40 eV) and UV spectra of libertellenone M

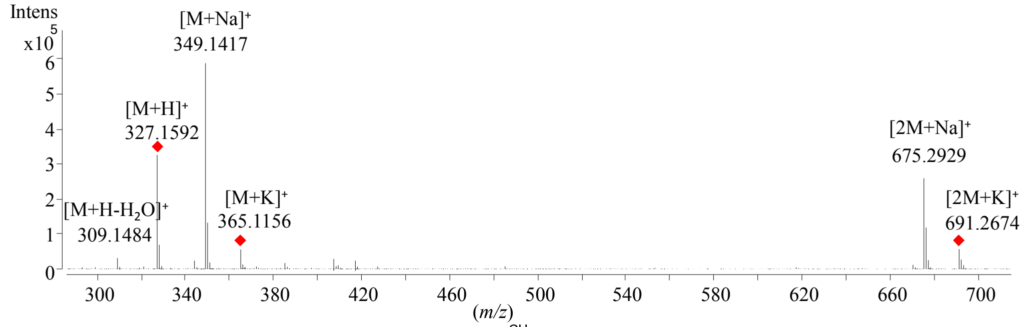


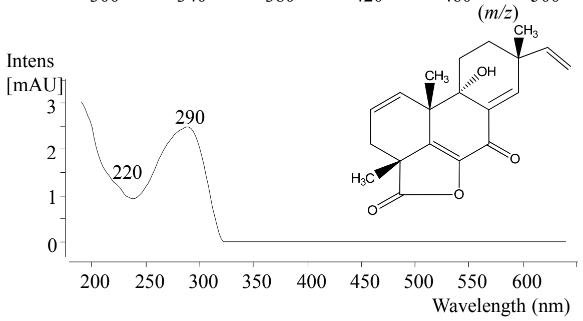


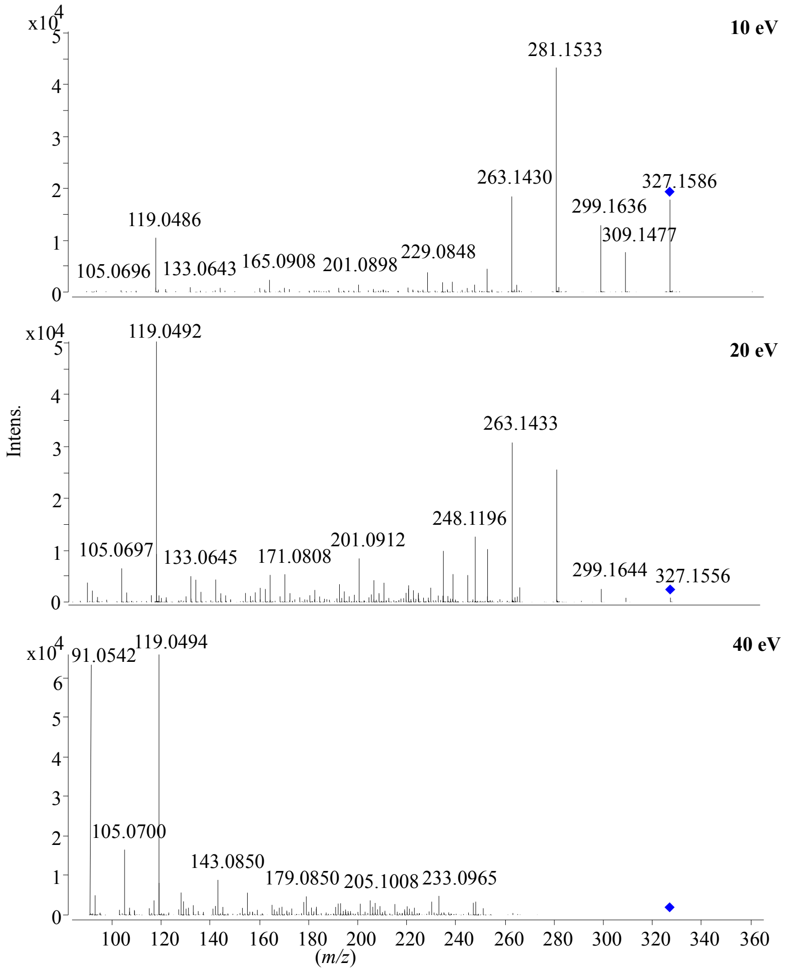

S3: MS, MS/HRMS (10, 20 and 40 eV) and UV spectra of opened γ-lactam libertellenone M

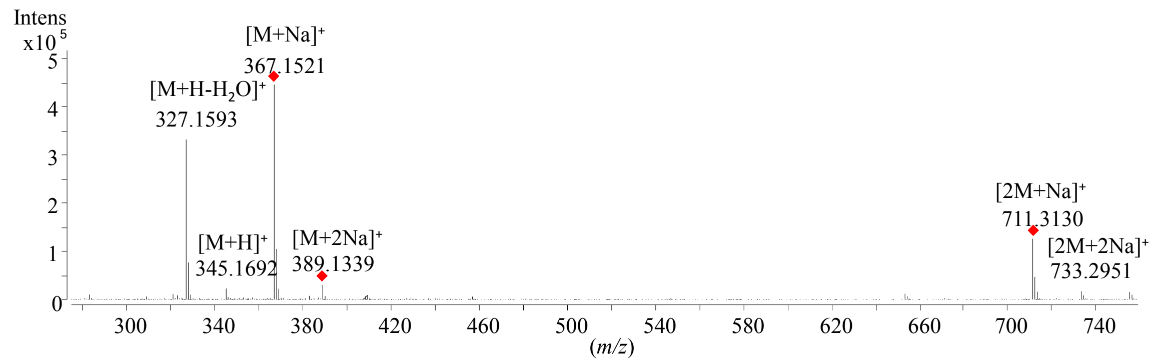


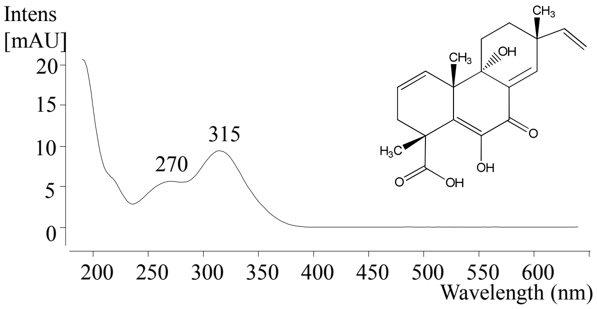


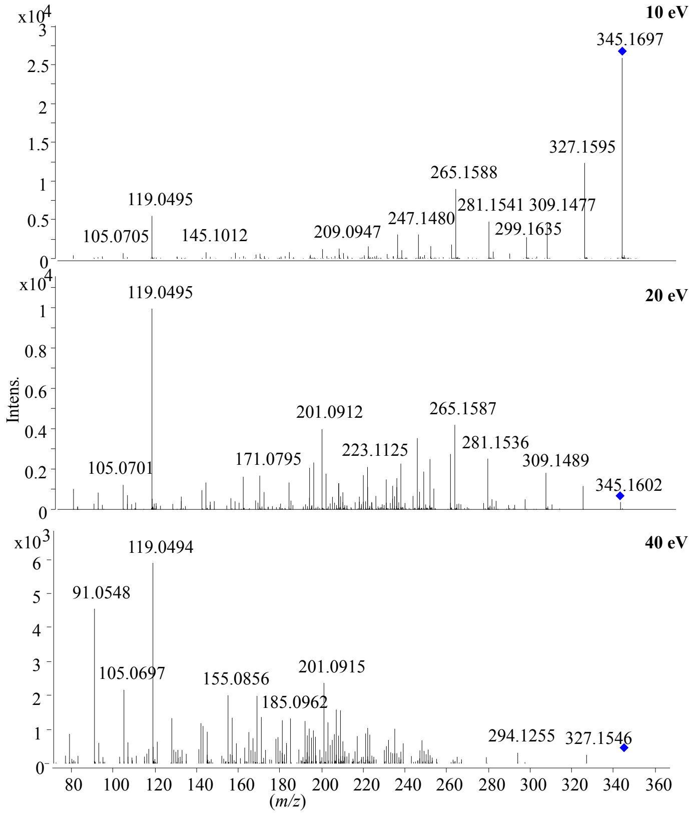

S4: BPC of the rice crude extract with EIC from HRMS showing the most abundant ion of the diterpenes: libertellenone E (*m/z* 369.1678 [M+Na]+), libertellenone C (*m/z* 331.1907 [M+H-$H_2O$]+), opened γ-lactone ring of libertellenone M libertellenone M (*m/z* 367.1512 [M+Na]+) and libertellenone M (*m/z* 349.1404 [M+Na]+).

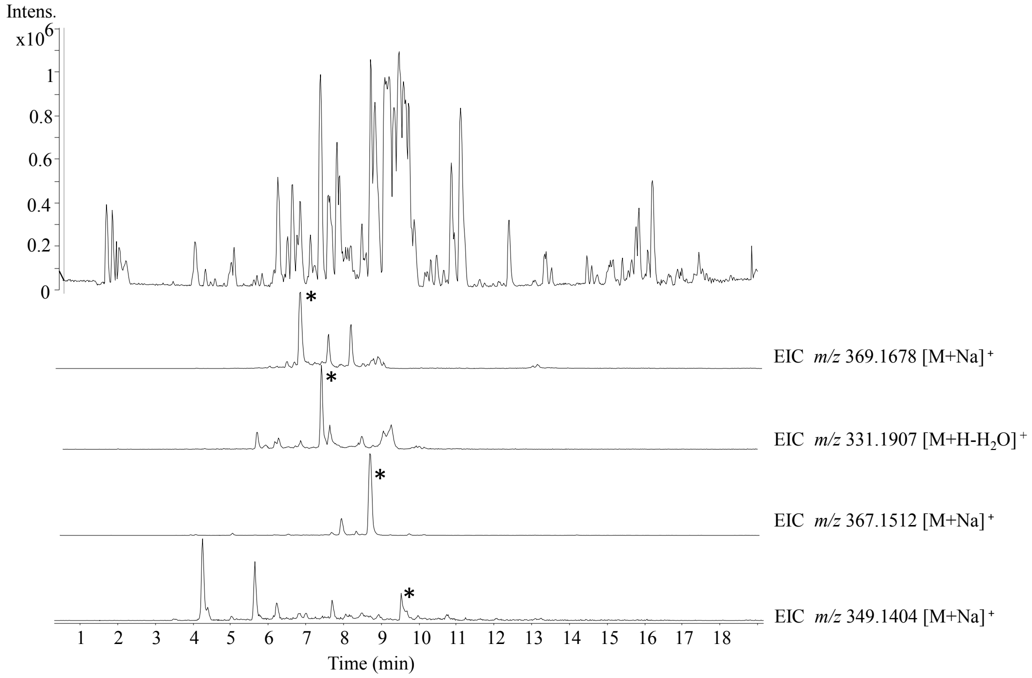


S5: BPC of the rice crude extract with EIC from MS/HRMS showing the fragment ion *m/z* 230.0451 and EIC from MS of *m/z* 450.2278, *m/z* 452.2436, *m/z* 420.2126 and *m/z* 434.2325 displaying ilicicolin H and the tentatively identified analogues and their position in the chromatogram

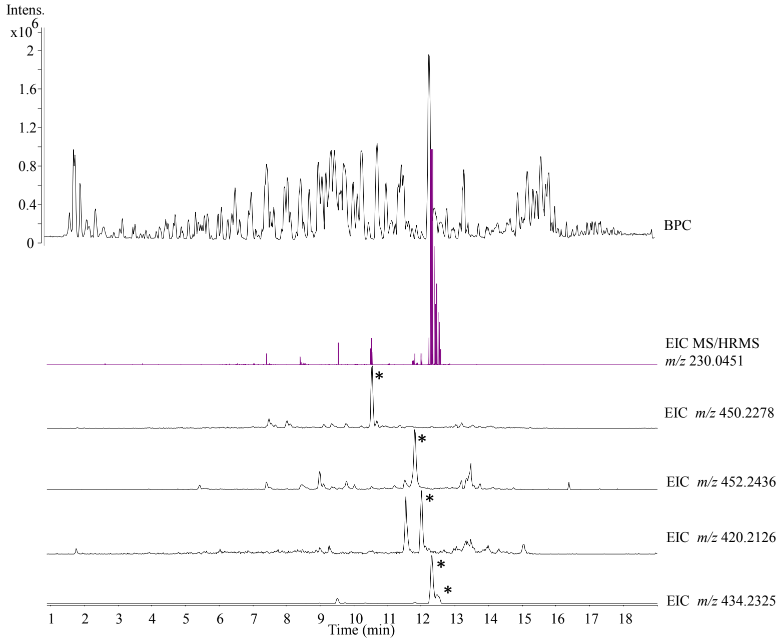

S6: MS/HRMS spectra, 40 eV of *m/z* 450.2278, *m/z* 452.2436, *m/z* 420.2126 and *m/z* 434.2325 compared to the library spectrum of ilicicolin H. UV spectrum of ilicicolin H.

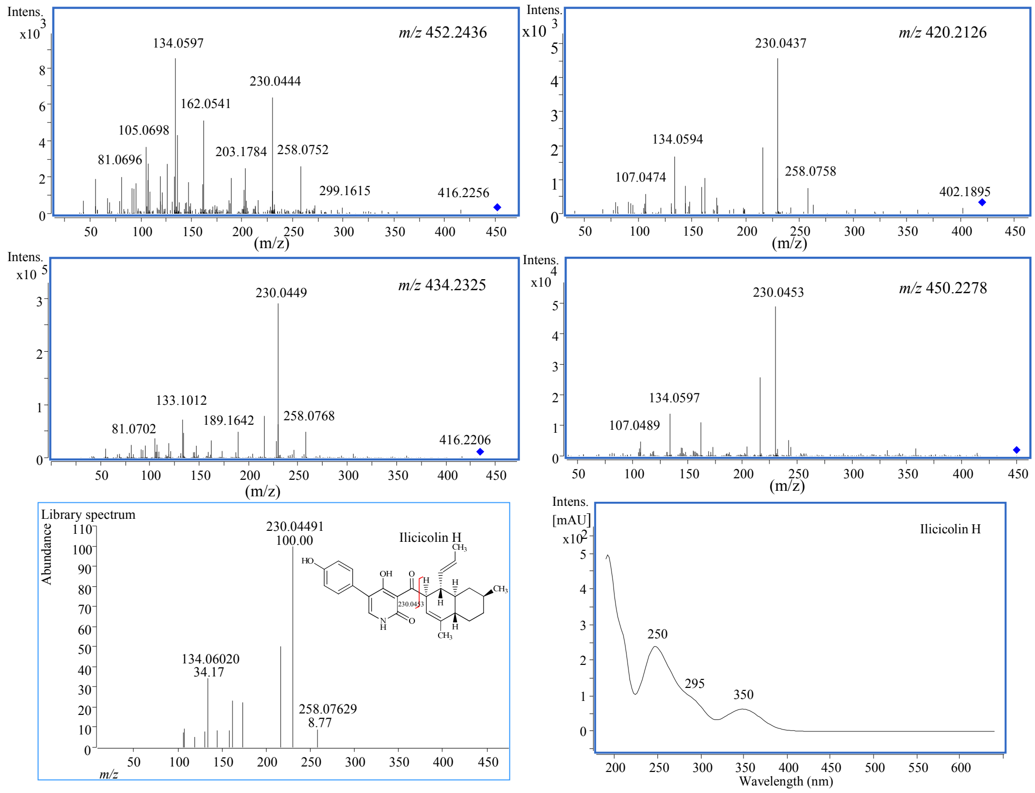


S7: MS and UV spectra of hydroxyl ilicicolin H ($C_{27}H_{31}NO_5$)

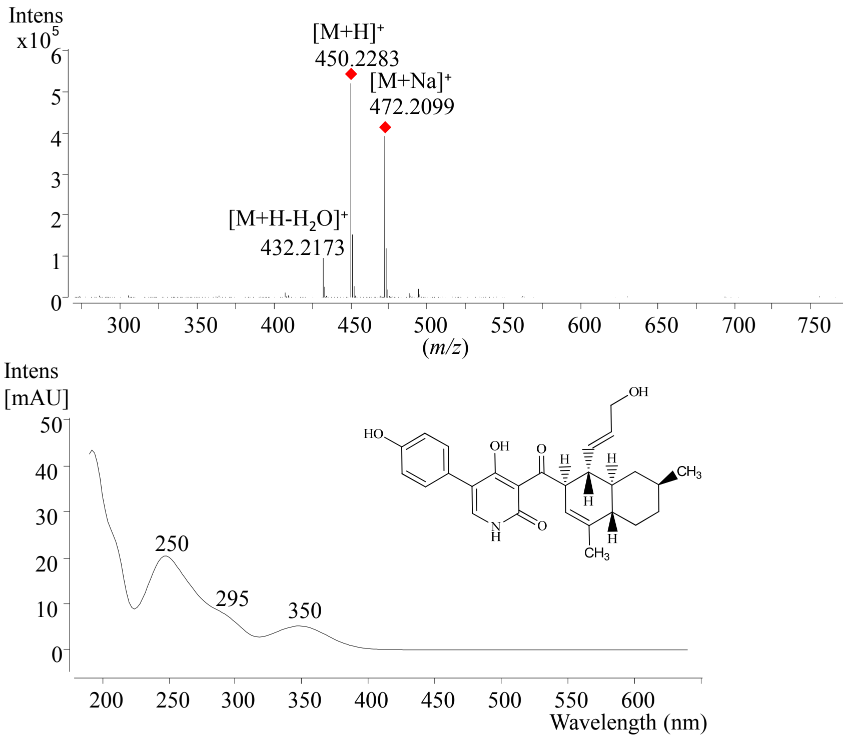

S8: MS and UV spectra of ilicicolin I ($C_{27}H_{31}NO_4$)

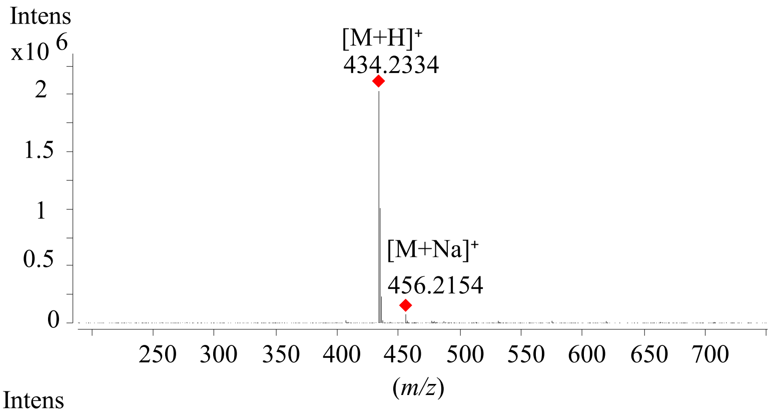


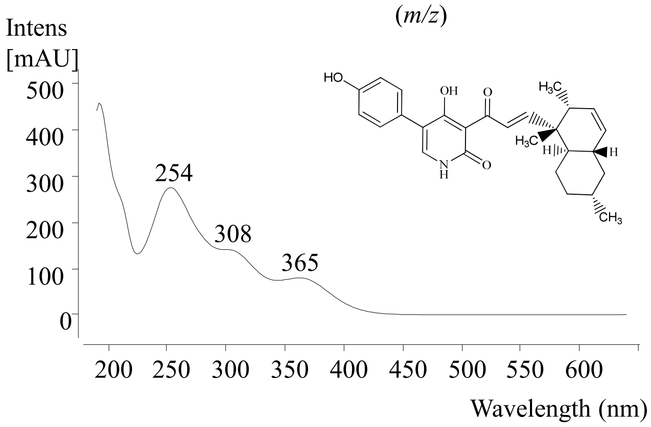

S9: $^{1}$H NMR (600 MHz CDCl3) of helvolic acid

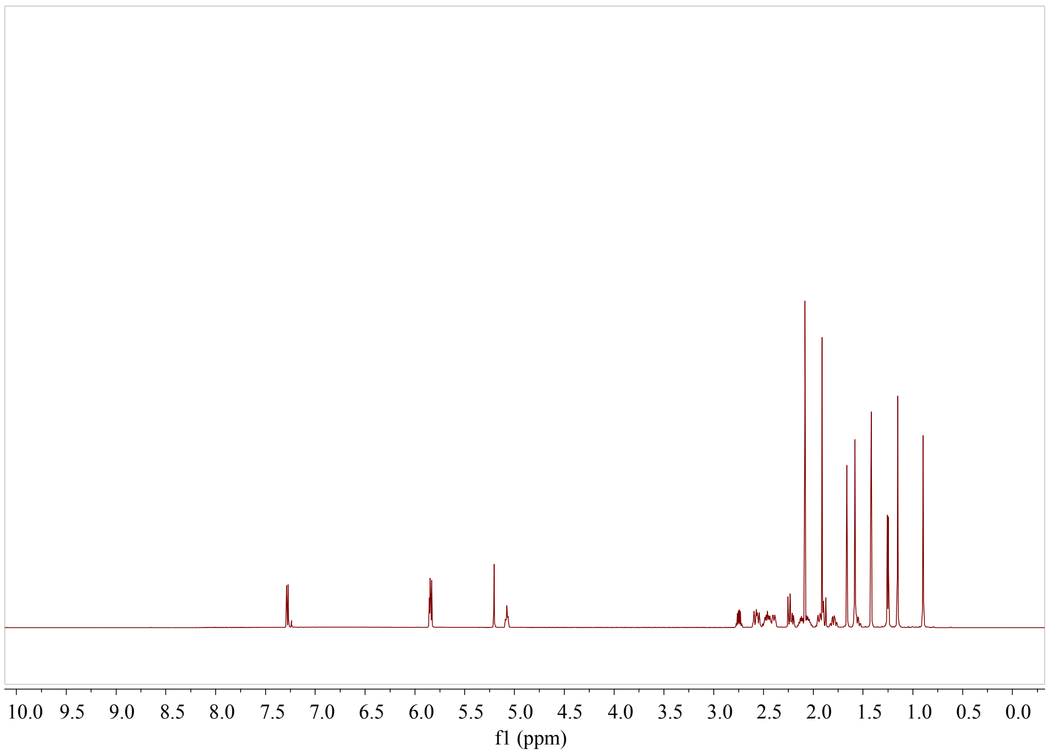


S10: $^{13}$C NMR (600 MHz CDCl3 ) of helvolic acid

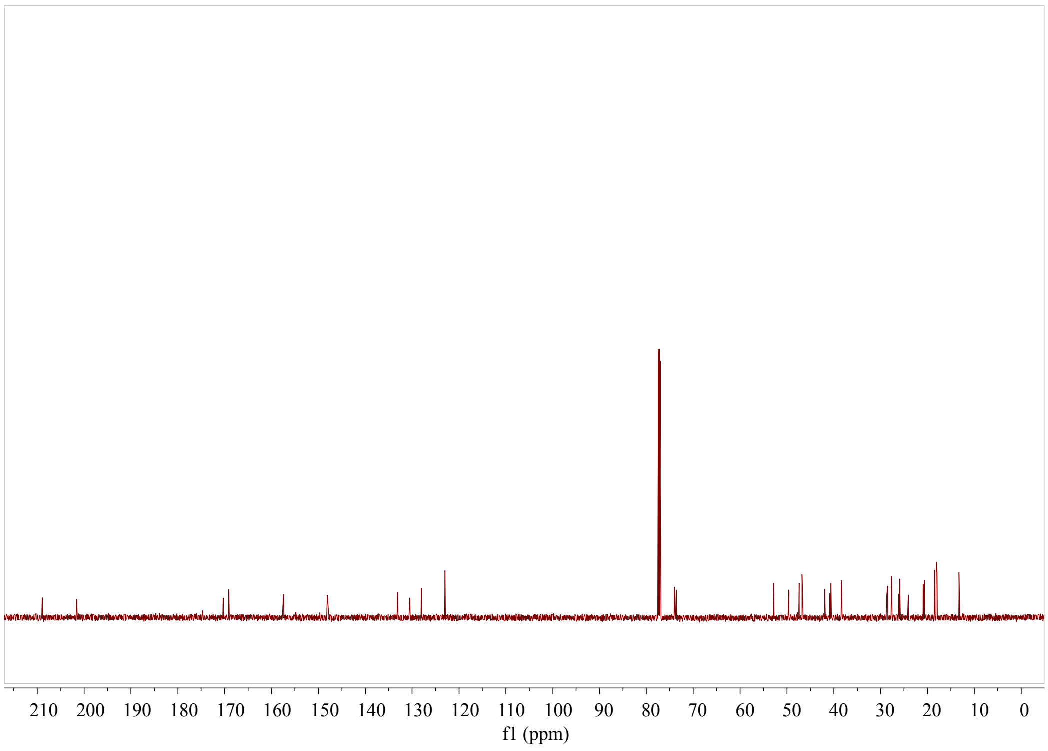

S11: Table 1. $^{13}$ C-NMR Spectroscopic Data (600 MHz $CD_3Cl$, δ in ppm) for helvolic acid

| Position | δ $^{13}$C |
|---|---|
| **1** | 157.5 |
| **2** | 128.0 |
| **3** | 201.6 |
| **4** | 40.9 |
| **5** | 47.4 |
| **6** | 73.7 |
| **7** | 209.0 |
| **8** | 52.9 |
| **9** | 41.9 |
| **10** | 38.4 |
| **11** | 24.1 |
| **12** | 26.1 |
| **13** | 49.6 |
| **14** | 46.8 |
| **15** | 40.6 |
| **16** | 74.0 |
| **17** | 148.1 |
| **18** | 18.1 |
| **19** | 27.7 |
| **20** | 130.5 |
| **21** | 174.7 |
| **22** | 28.7 |
| **23** | 28.5 |
| **24** | 122.9 |
| **25** | 133.1 |
| **26** | 17.9 |
| **27** | 26.0 |
| **28** | 13.3 |
| **29** | 18.5 |
| **30/32** | 169.1/170.3 |
| **31/33** | 20.9/20.7 |

S12: $^{1}$H-NMR 400 MHz MeOD of myrocin F

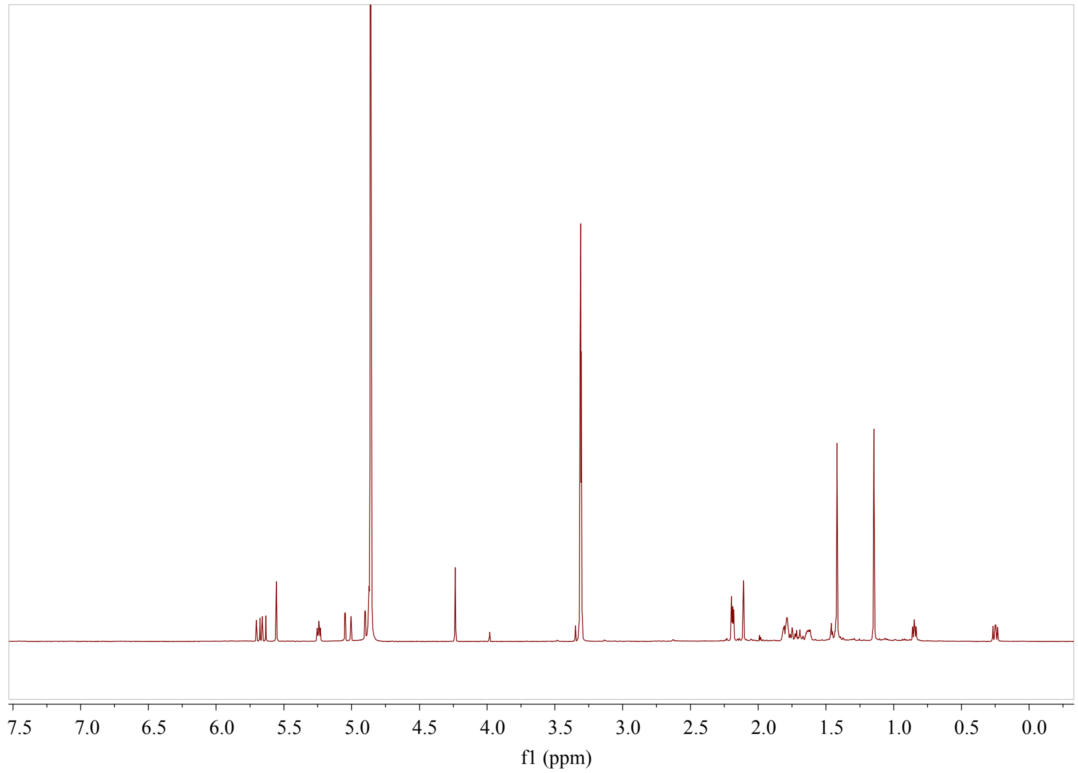


S13: $^{13}$C NMR 400 MHz MeOD of myrocin F

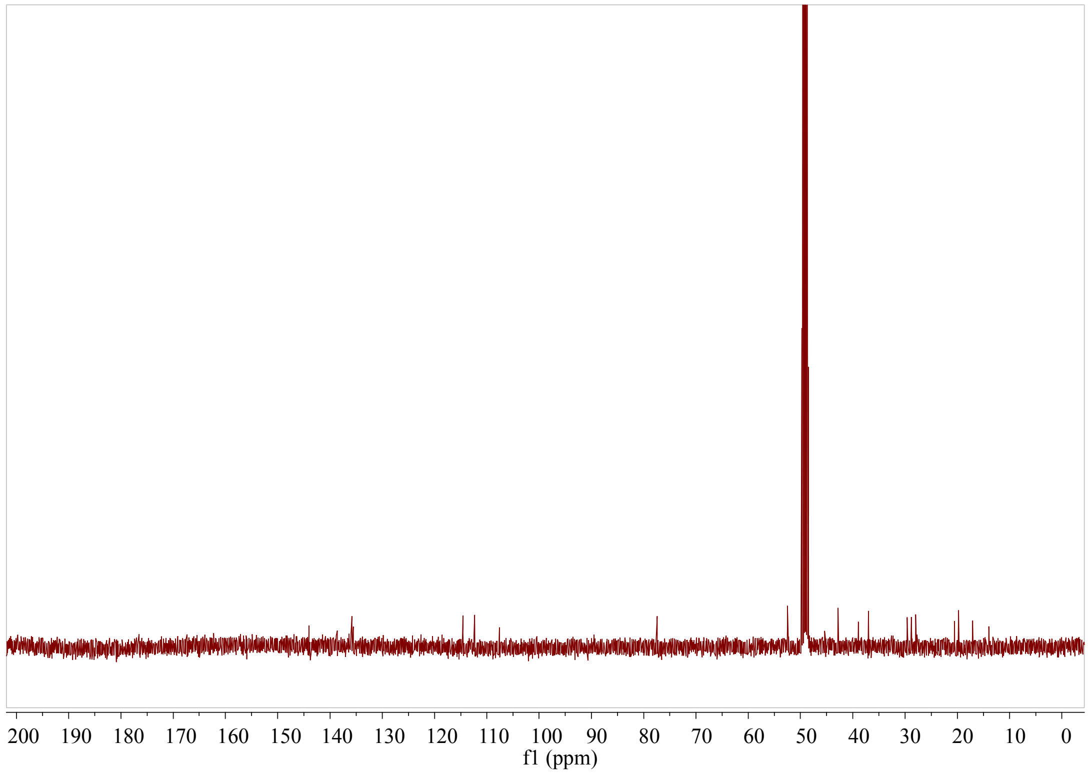

S14: COSY spectrum (400 MHz MeOD) of myrocin F

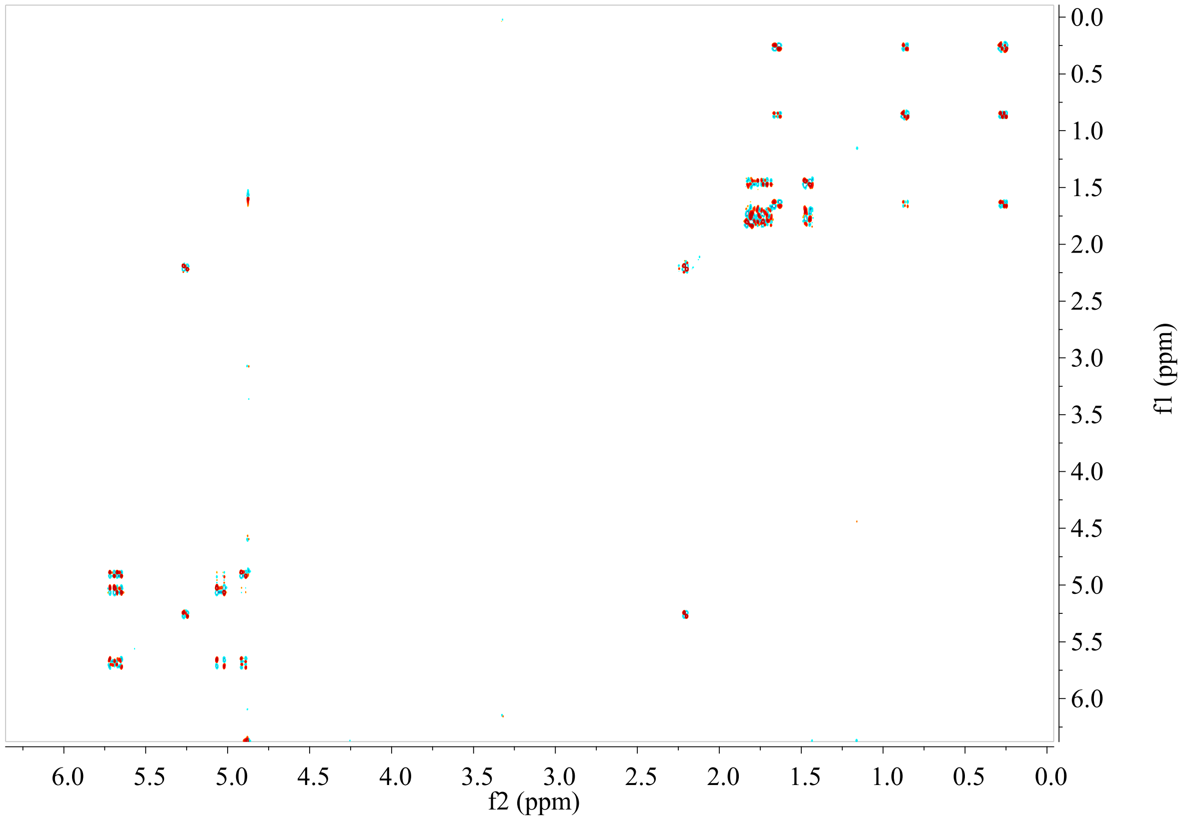


S15: Edited HSQC spectrum (400 MHz MeOD) of myrocin F

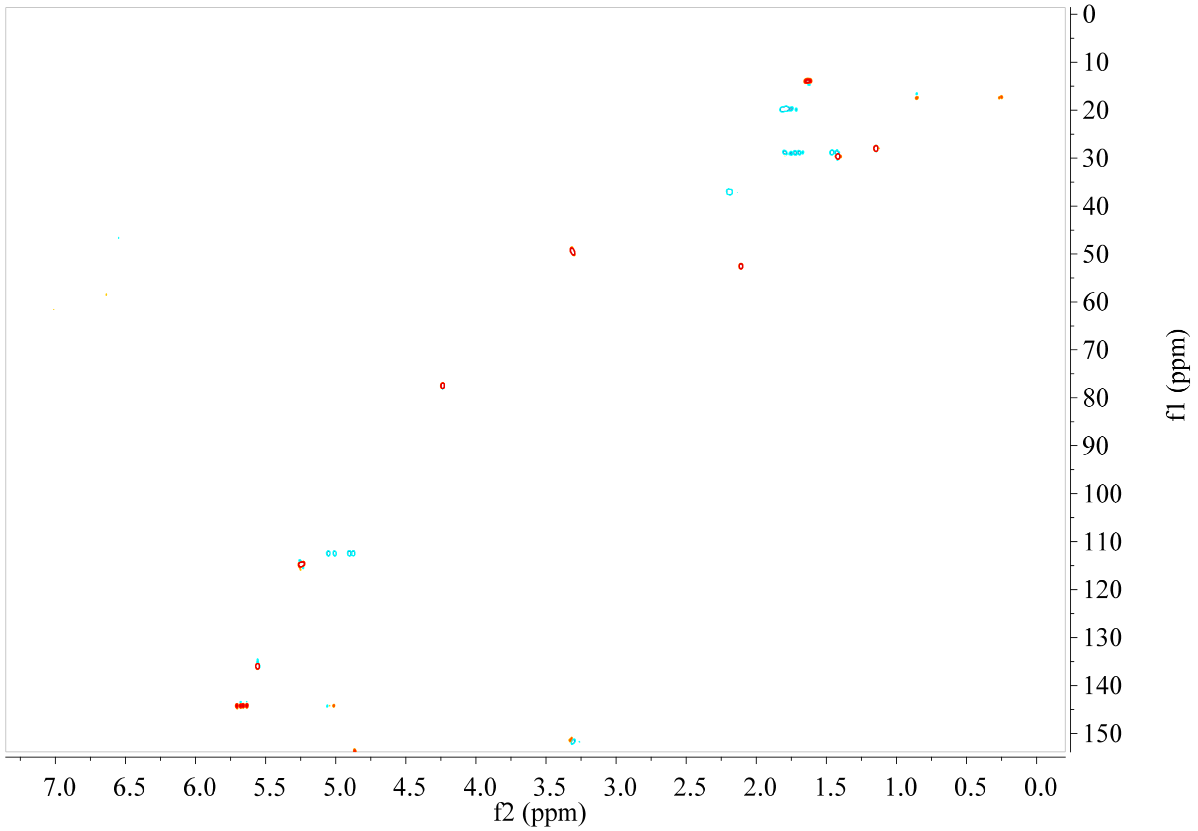

S16: HMBC spectrum (400 MHz MeOD) of myrocin F

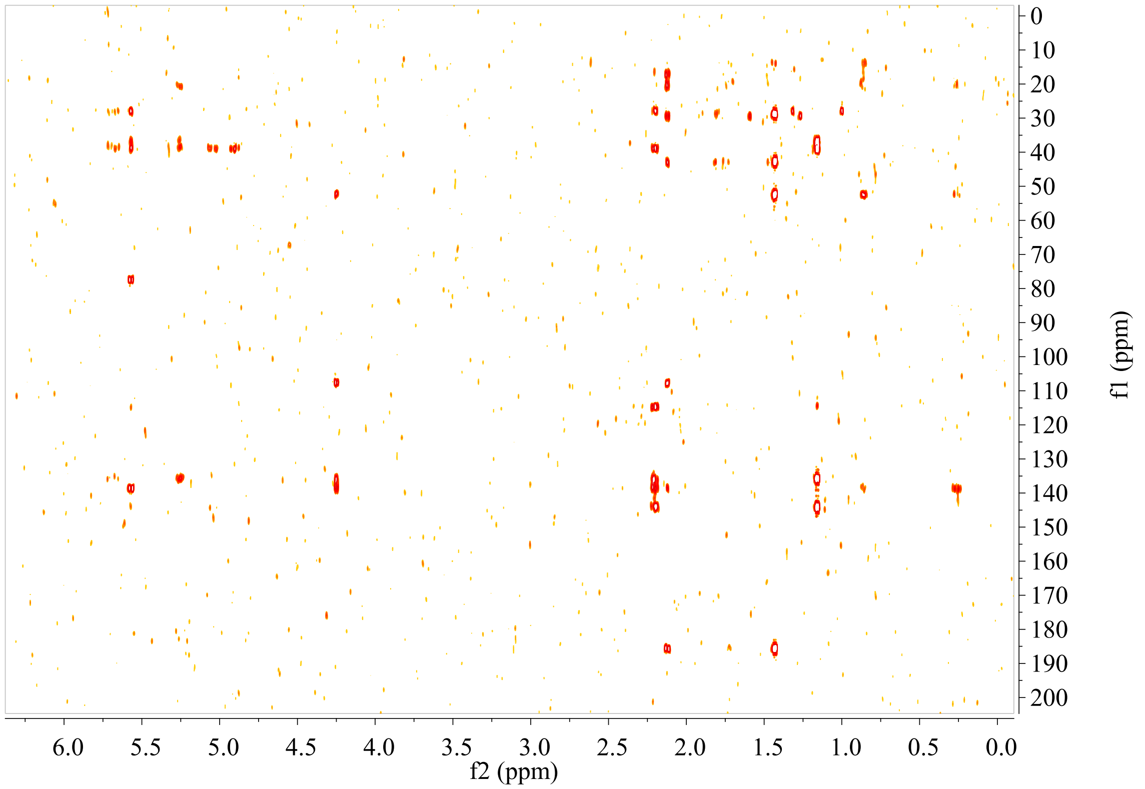


S17: NOESY spectrum (400 MHz MeOD) of myrocin F

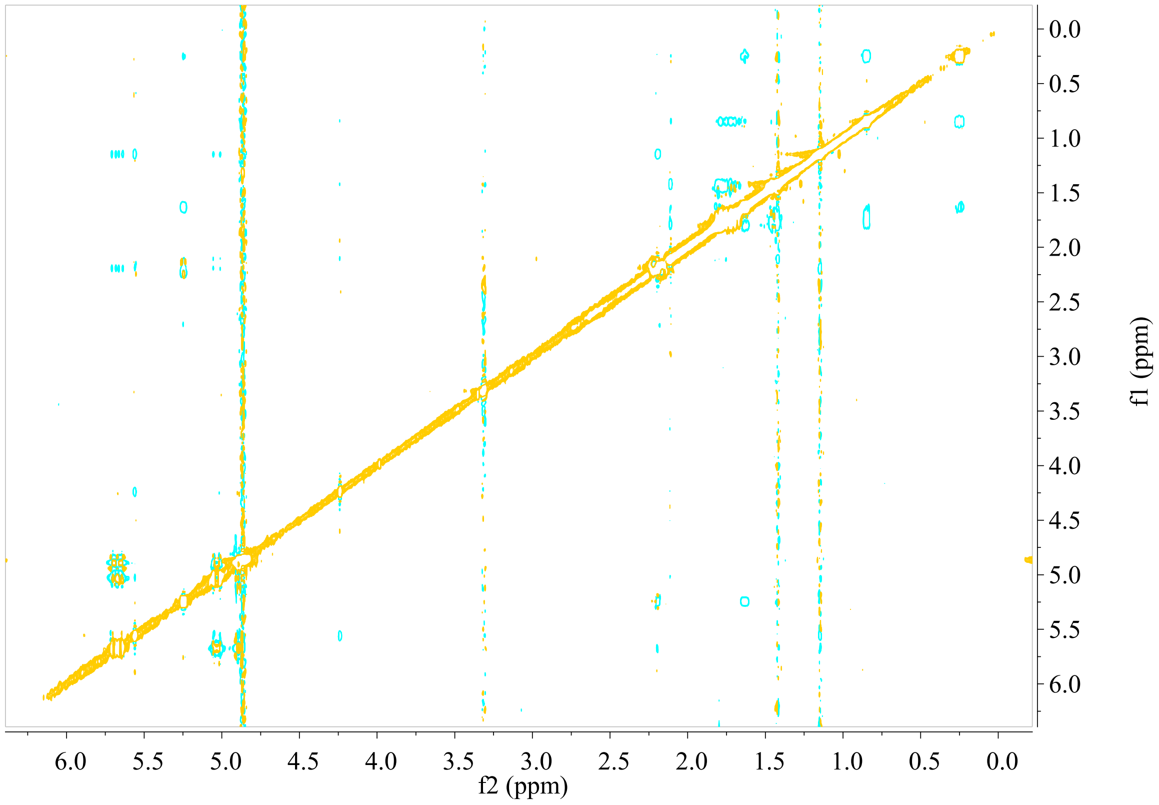

S18: $^1$H-NMR (800 MHz $CD_3CN$) of libertellenone M

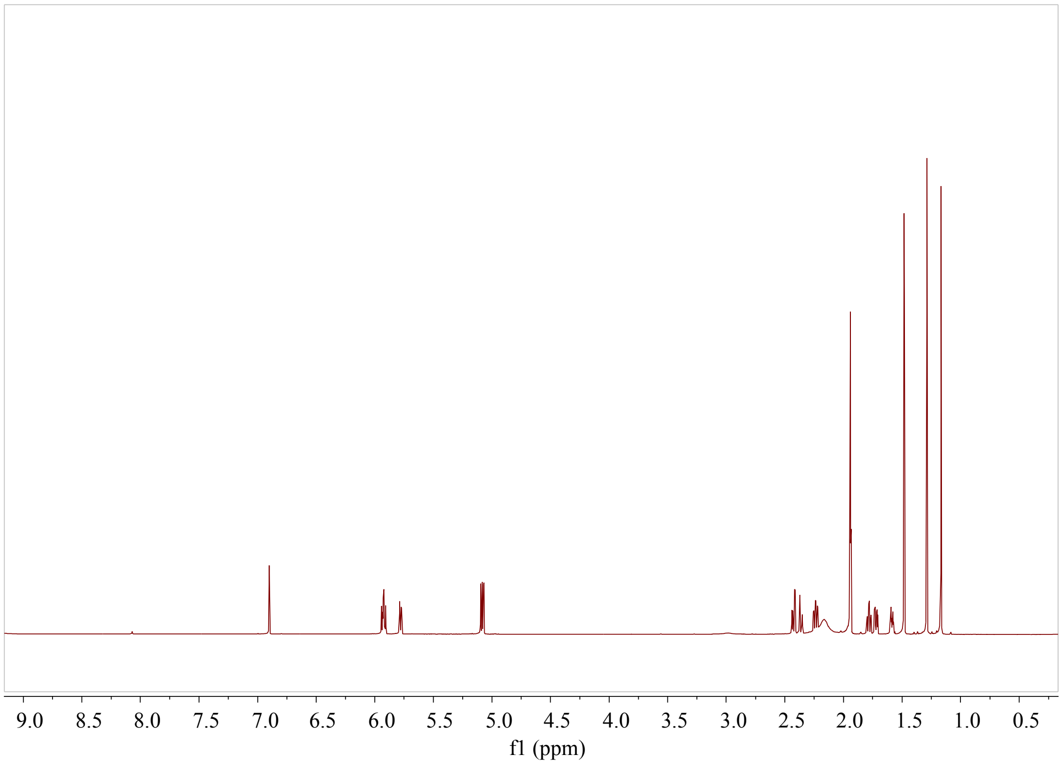


S19: $^{13}$C-NMR (800 MHz $CD_3CN$) of libertellenone M

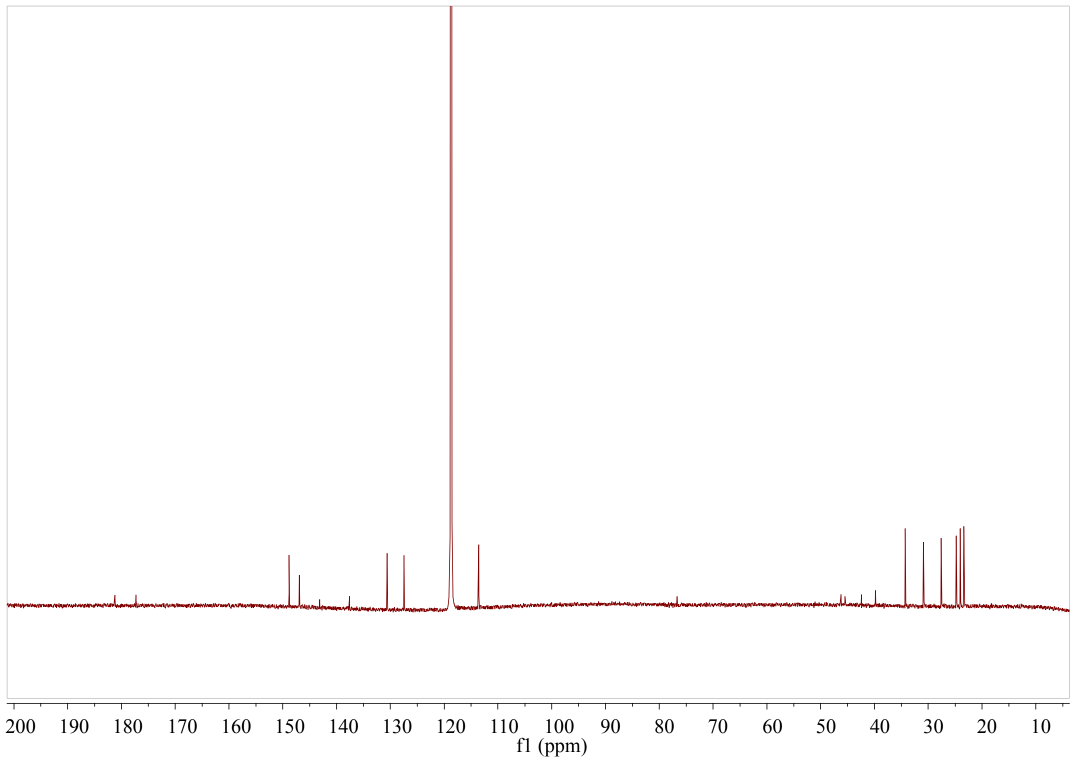

S20: DQF-COSY spectrum (800 MHz $CD_3CN$) of libertellenone M

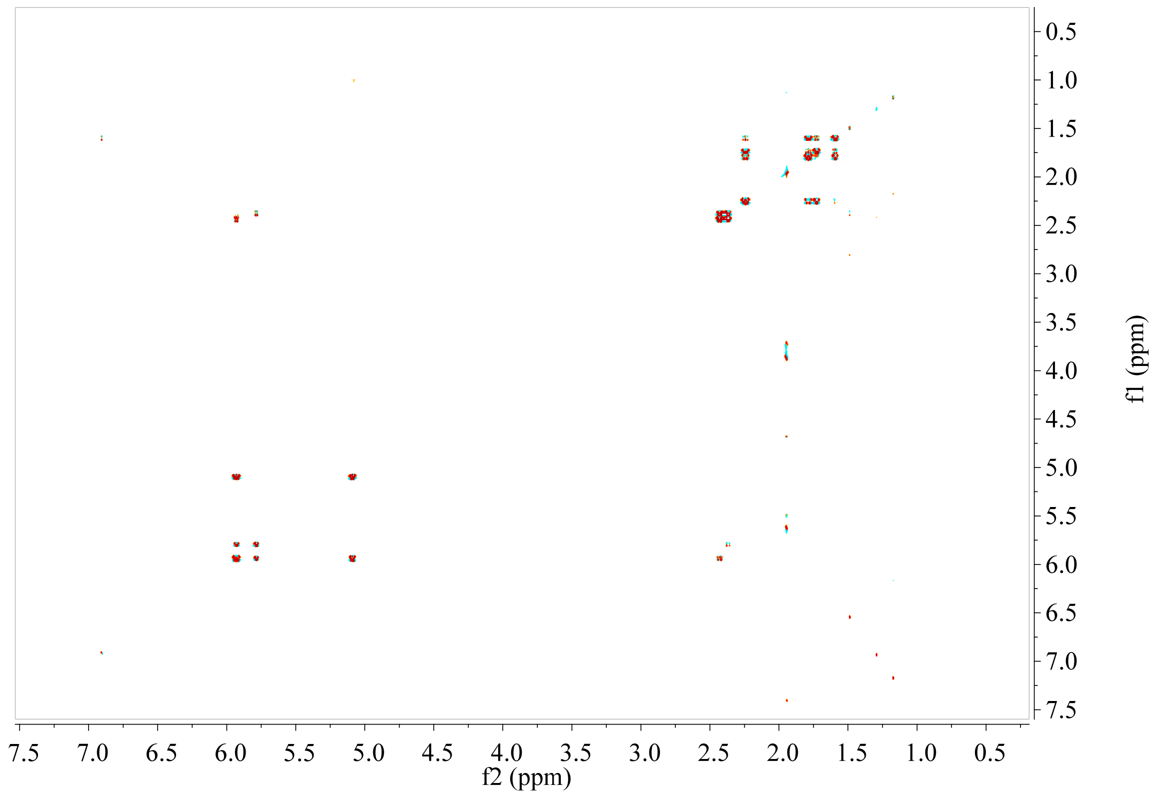


S21: Edited HSQC (800 MHz $CD_3CN$) of libertellenone M

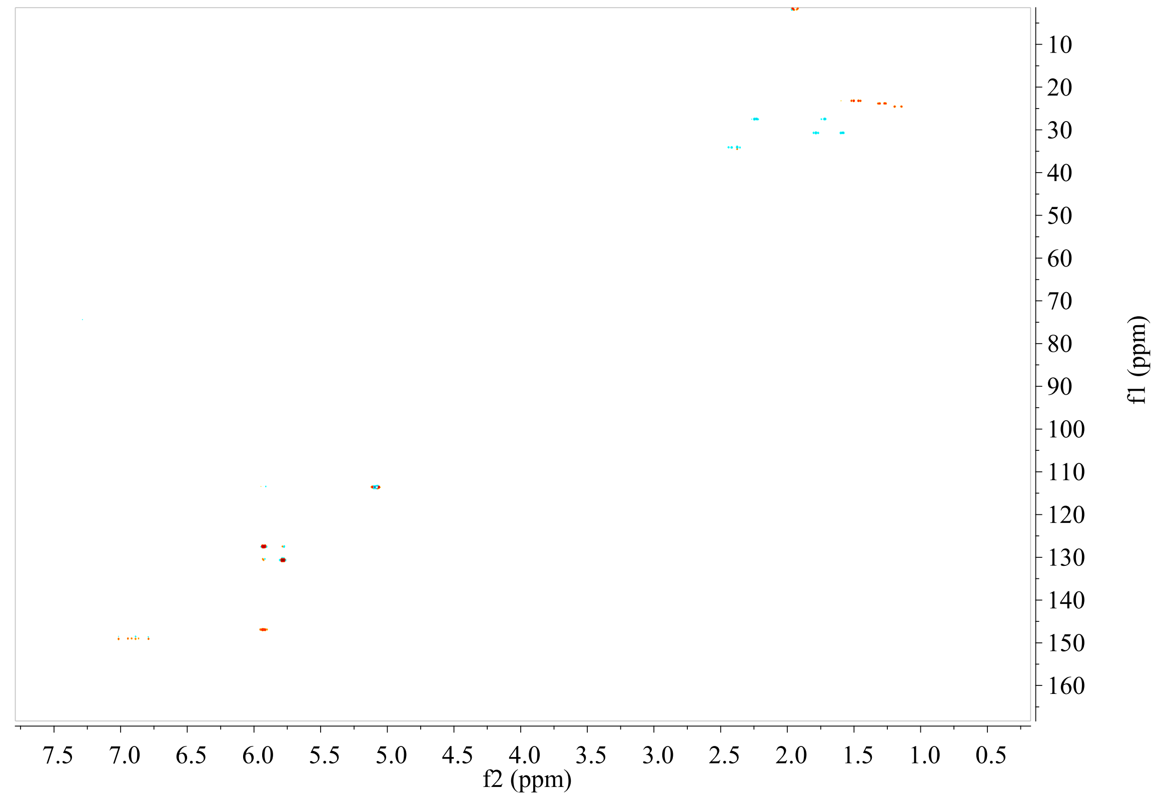

S22: HMBC spectrum (800 MHz CD3CN) of libertellenone M

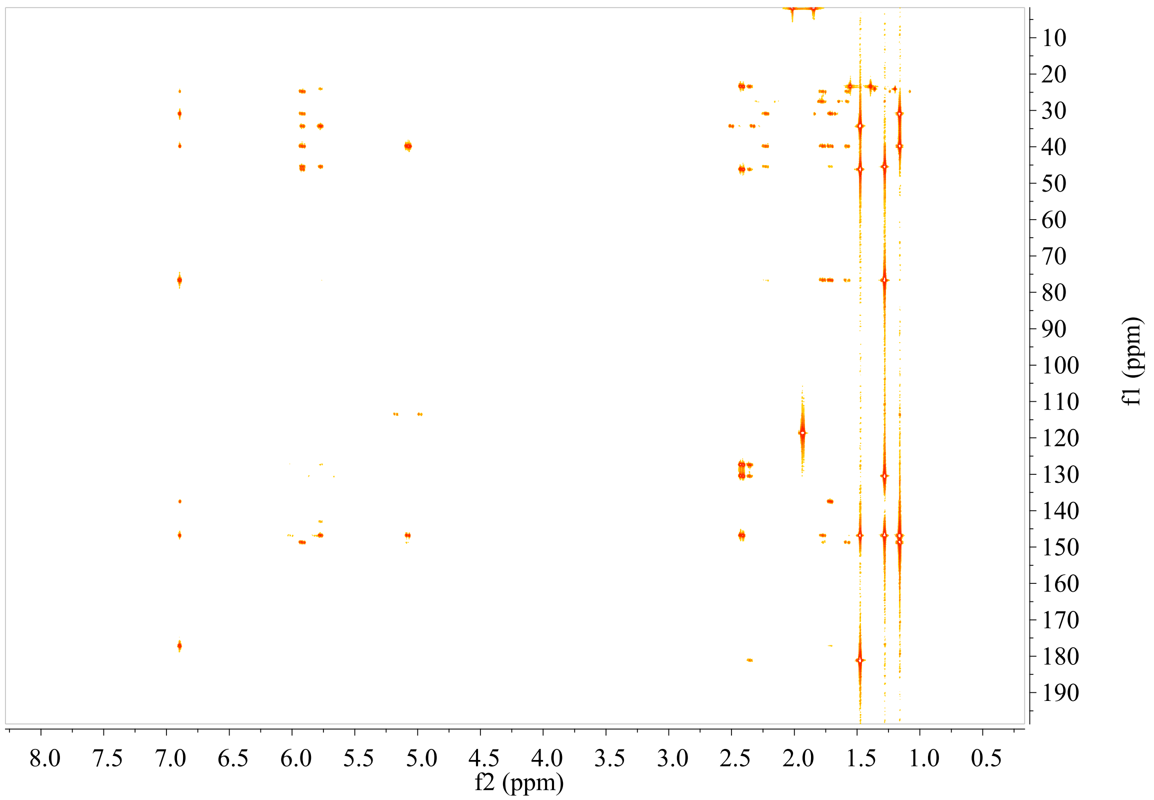


S23: NOESY spectrum (400 MHz CD3CN) of libertellenone M

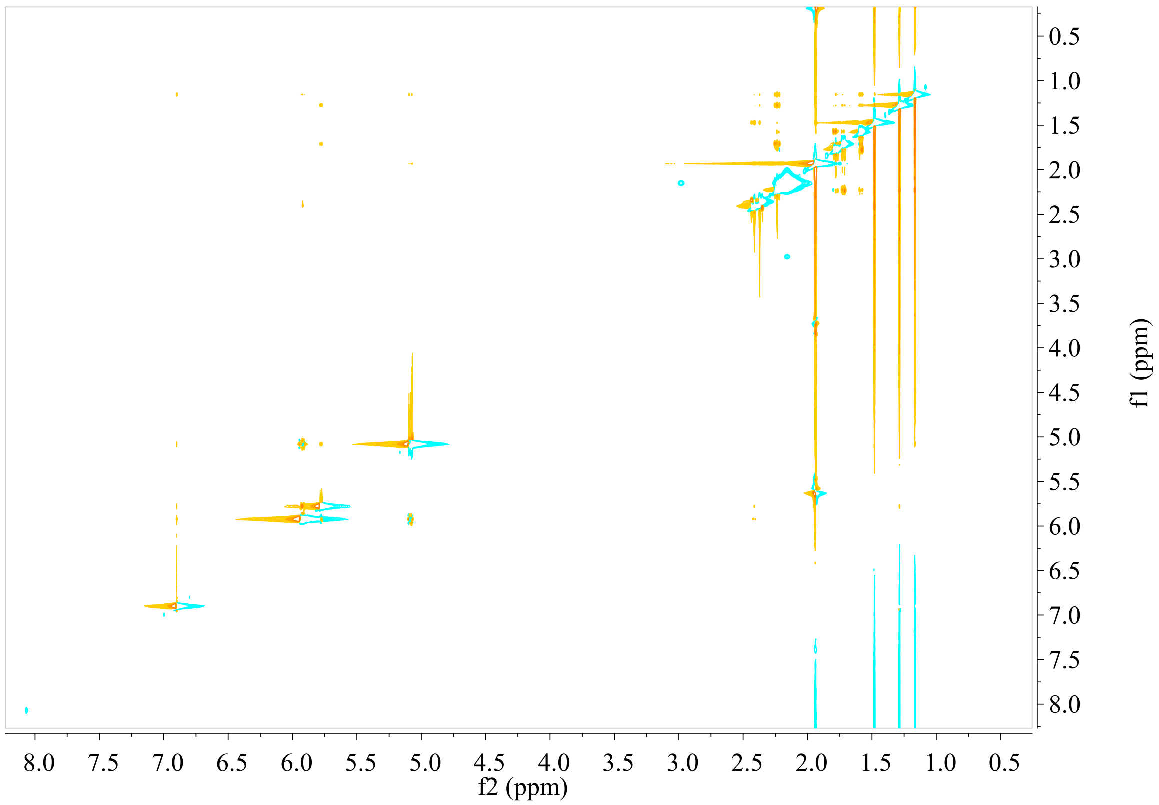

S24: $^{1}$H-NMR (800 MHz MeOD) of opened γ-lactam libertellenone M

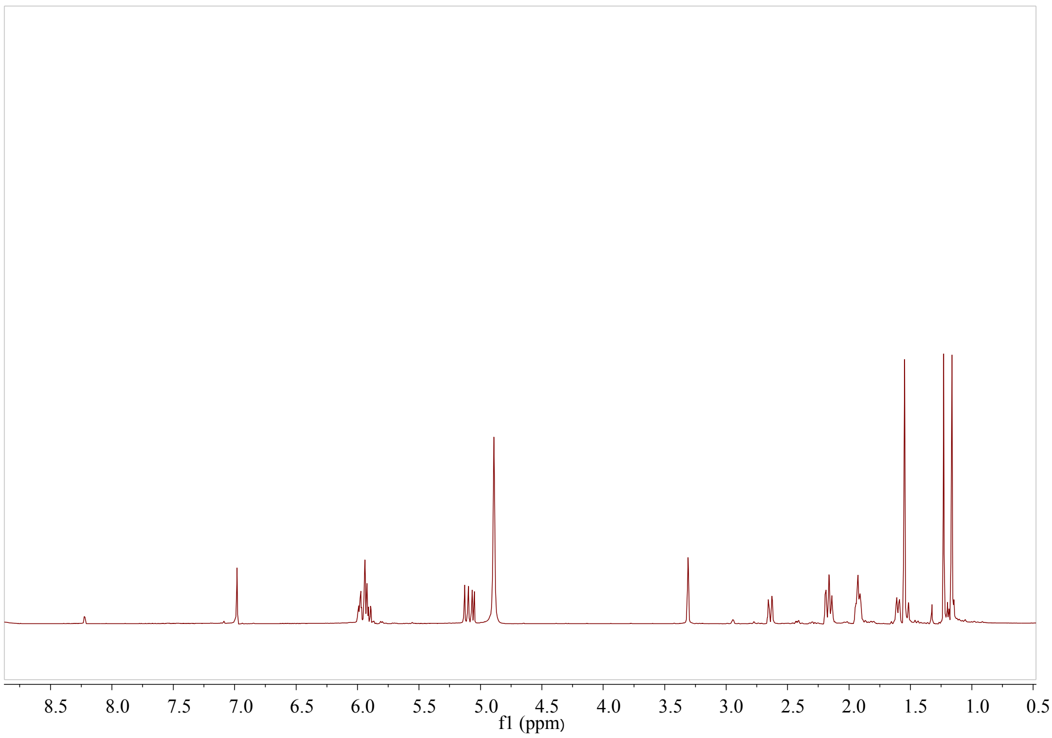


S25: DQF-COSY (800 MHz MeOD) of opened γ-lactam libertellenone M

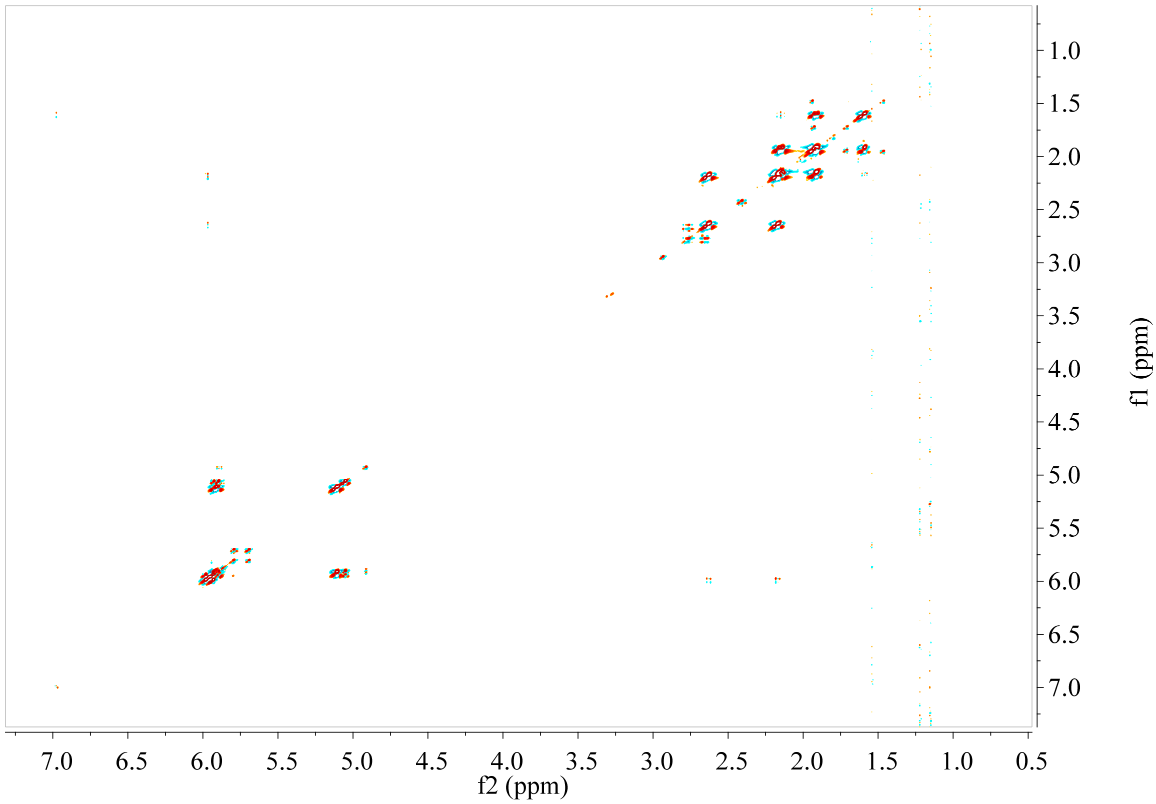

S26: Edited-HSQC (800 MHz MeOD) of opened γ-lactam libertellenone M

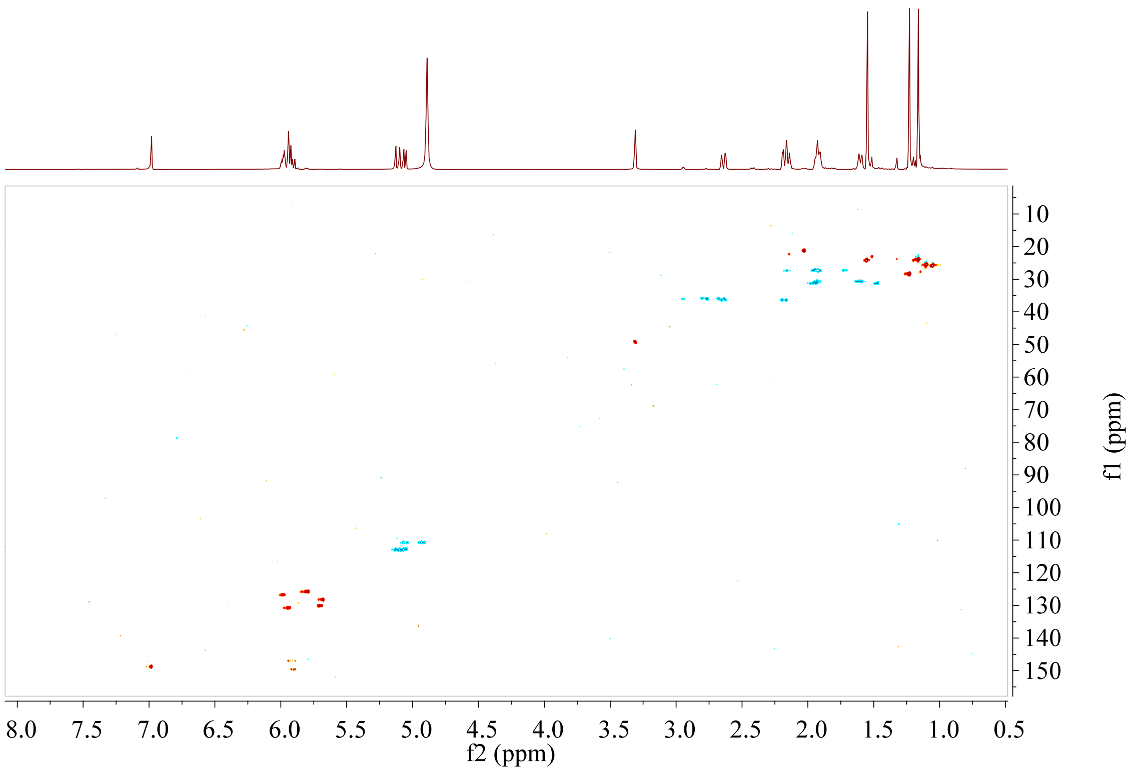


S27: HMBC (800 MHz MeOD) of opened γ-lactam libertellenone M

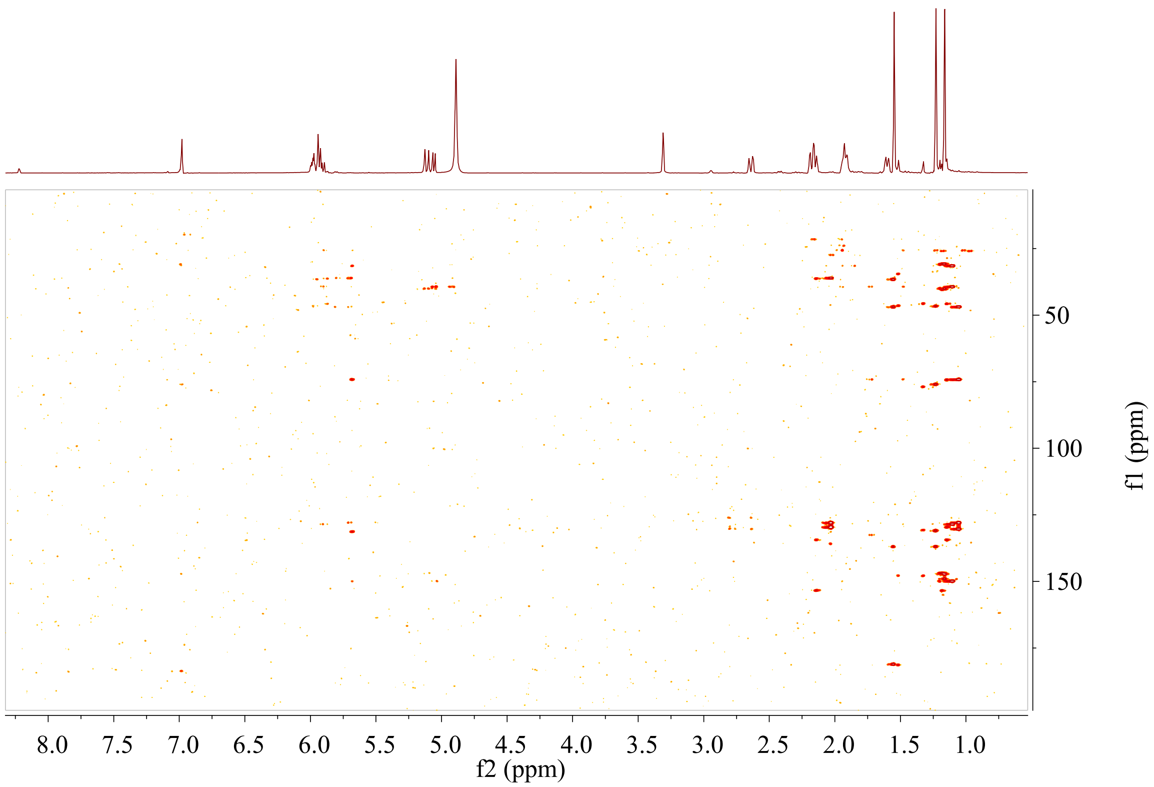

S28: $^{1}$H-NMR (800 MHz $CD_3CN$) of libertellenone C

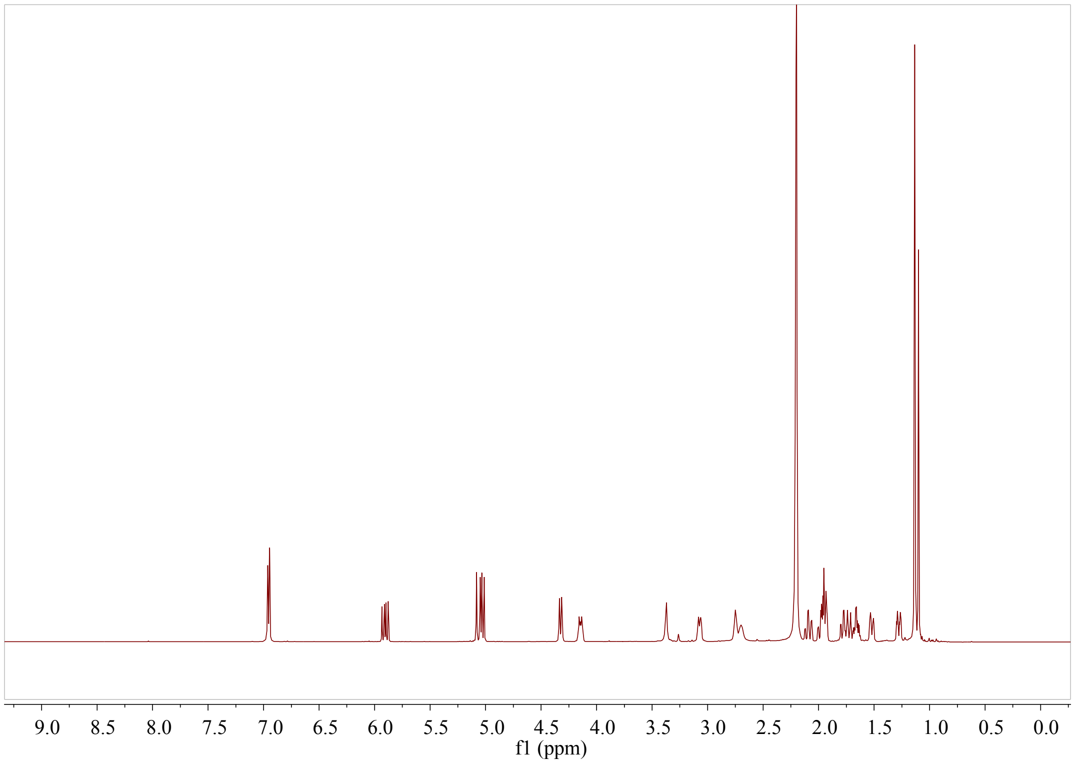


S29: $^{13}$C-NMR (800 MHz $CD_3CN$) of libertellenone C

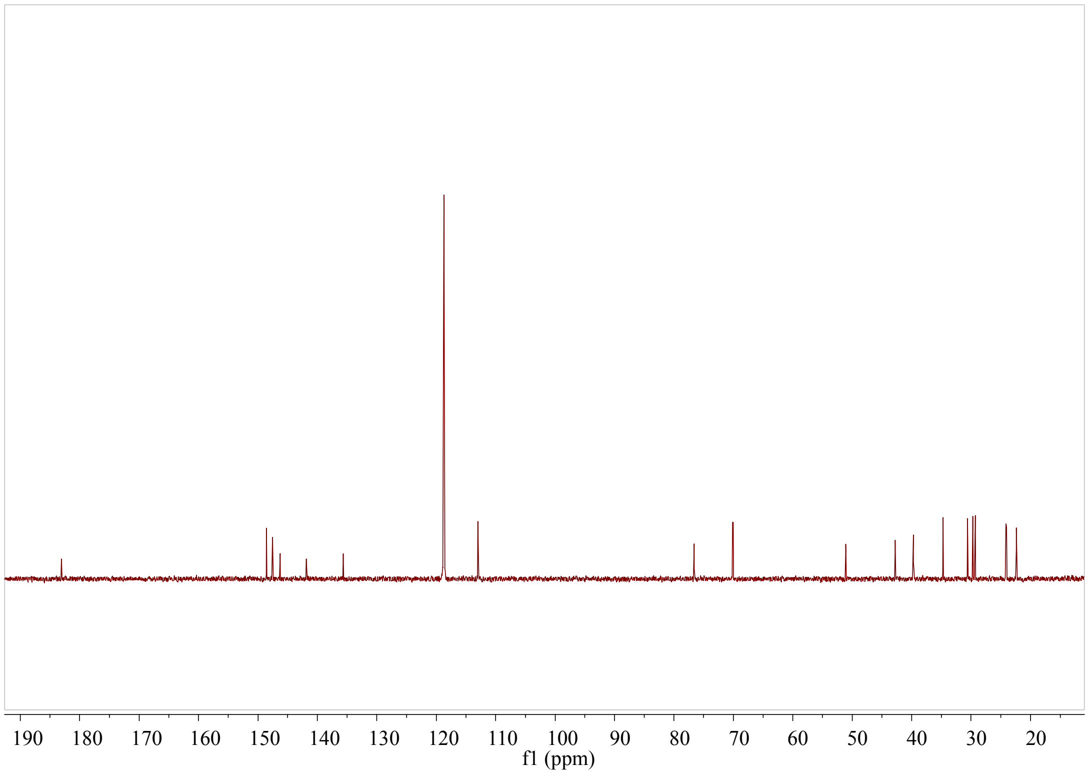

S30: $^{1}$H-NMR (800 MHz $CD_3CN$) of libertellenone E

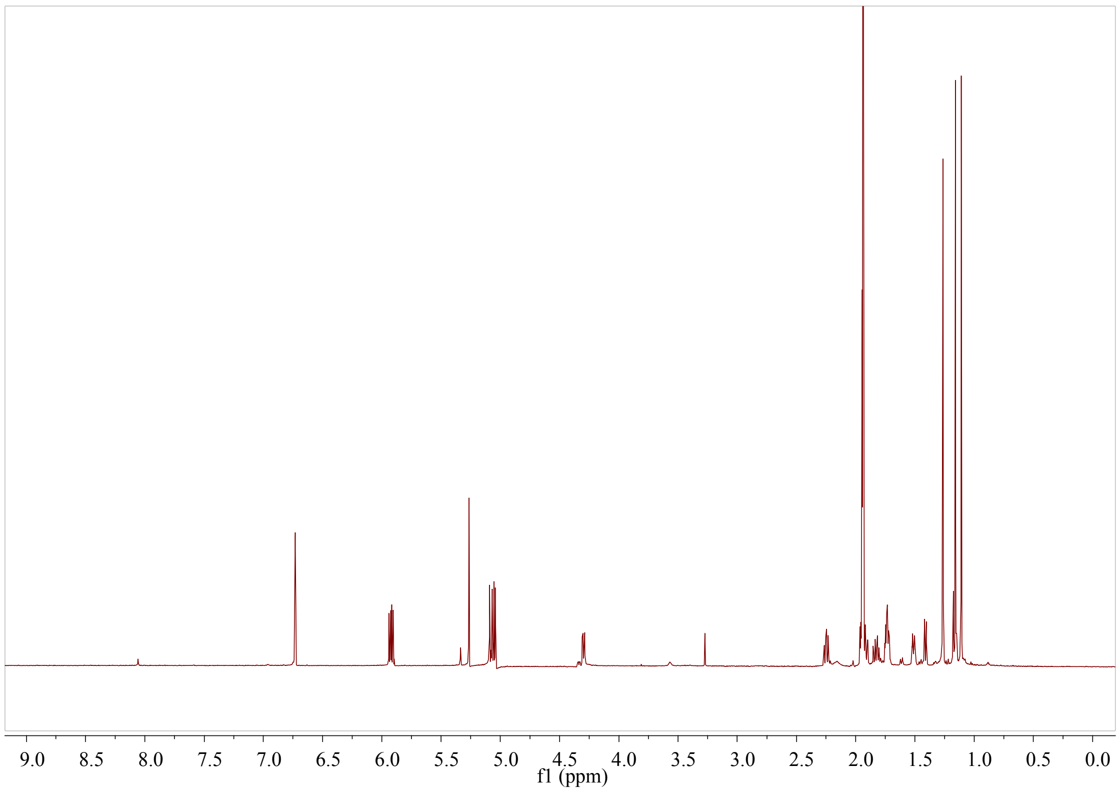

S31: Table 2. NMR Spectroscopic Data (400 MHz, MeCN-d3, δ in ppm, *J* in Hz) for libertellenone C and libertellenone E.

| | Libertellenone C | | Libertellenone E | |
|---|---|---|---|---|
| | δ $^{13}$C | δ $^{1}$H (mult, *J*) | δ $^{13}$C | δ $^{1}$H (mult, *J*) |
| **1** | 70.1 | 4.15 m | 69.8 | 4.30 dd(11.6,4.5) |
| **2a** | 29.3 | 1.66 m | 29.2 | 1.73 m |
| **2b** | | 1.72 m | | 1.81 m |
| **3a** | 34.7 | 1.28 m | 27.4 | 1.41 dt(13.4,3.5) |
| **3b** | | 1.98 m | | 1.91 dd(13.5,3.5) |
| **4** | 42.8 | - | 49.7 | - |
| **5** | 141.9 | - | 146.7 | - |
| **6** | 146.3 | - | 146.0 | - |
| **7** | 183.0 | - | 179.6 | - |
| **8** | 135.7 | - | 138.5 | - |
| **9** | 76.6 | - | 75.9 | - |
| **10** | 51.1 | - | 48.4 | - |
| **11a** | 29.7 | 2.09 td(14.4,3.3) | 29.3 | 2.25 td(14.4,3.4) |
| **11b** | | 1.96 m | | 1.96 m |
| **12a** | 30.6 | 1.52 m | 30.9 | 1.51 m |
| **12b** | | 1.78 m | | 1.74 m |
| **13** | 39.7 | - | 39.3 | - |
| **14** | 148.6 | 6.96 d(1.6) | 146.1 | 6.73 s |
| **15** | 147.6 | 5.90dd(17.6,10.7) | 147.7 | 5.92 dd(17.3,10.9) |
| **16a** | 113.0 | 5.07 d(17.6) | 113.0 | 5.08 d(17.3) |
| **16b** | | 5.02 d(10.7) | | 5.05 d(10.9) |
| **17** | 24.0 | 1.10 s | 24.4 | 1.11 s |
| **18** | 22.4 | 1.13s | 25.6 | 1.26 s |
| **19a** | 70.2 | 4.32 d(10.4) | 108.0 | 5.26 s |
| **19b** | | 3.07 d(10.4) | - | - |
| **20** | 24.1 | 1.13 s | 18.1 | 1.16 s |

S32: $^{1}$H-NMR (500 MHz $CD_3CN$) of hydroxyl-ilicicolin H

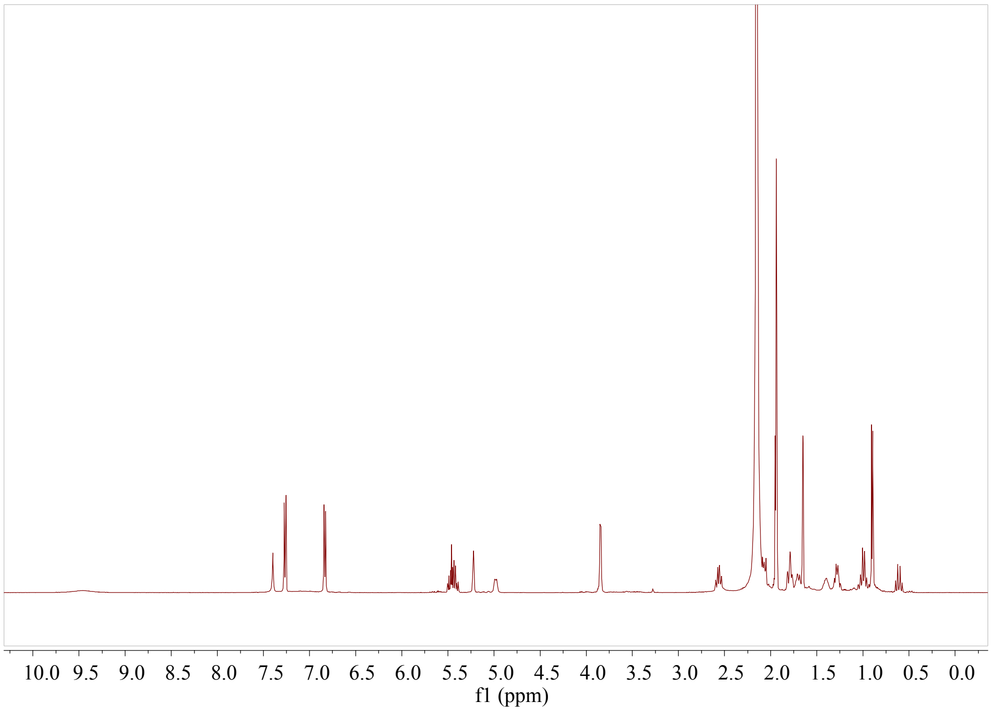


S33: $^{13}$C-NMR (500 MHz $CD_3CN$) of hydroxyl-ilicicolin H

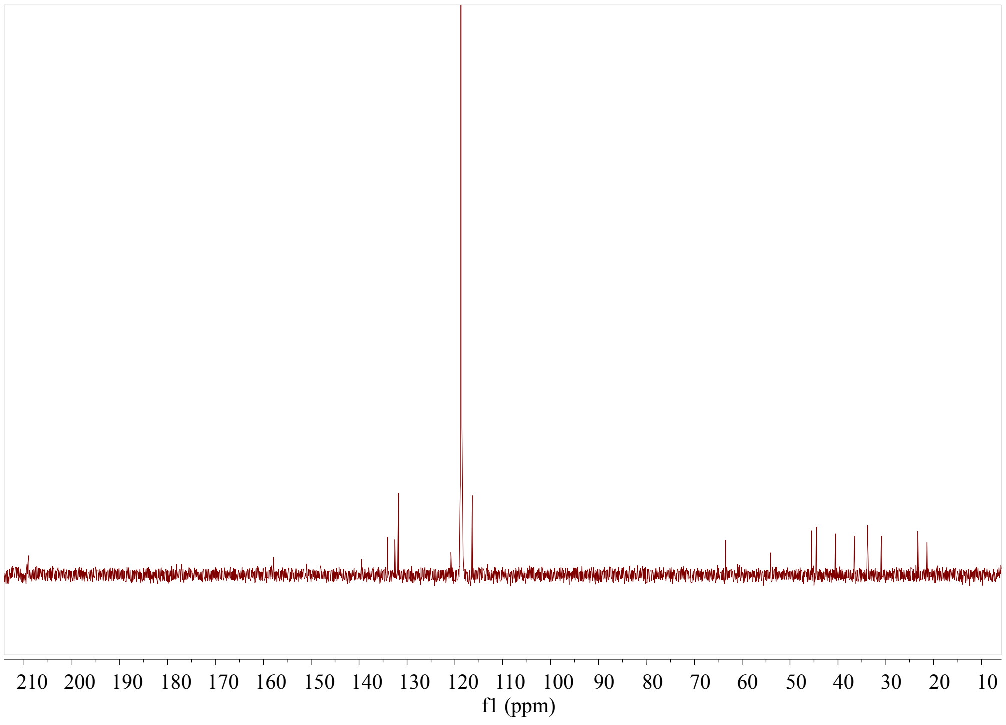

S34: COSY spectrum (500 MHz CD3CN) of hydroxyl-ilicicolin H

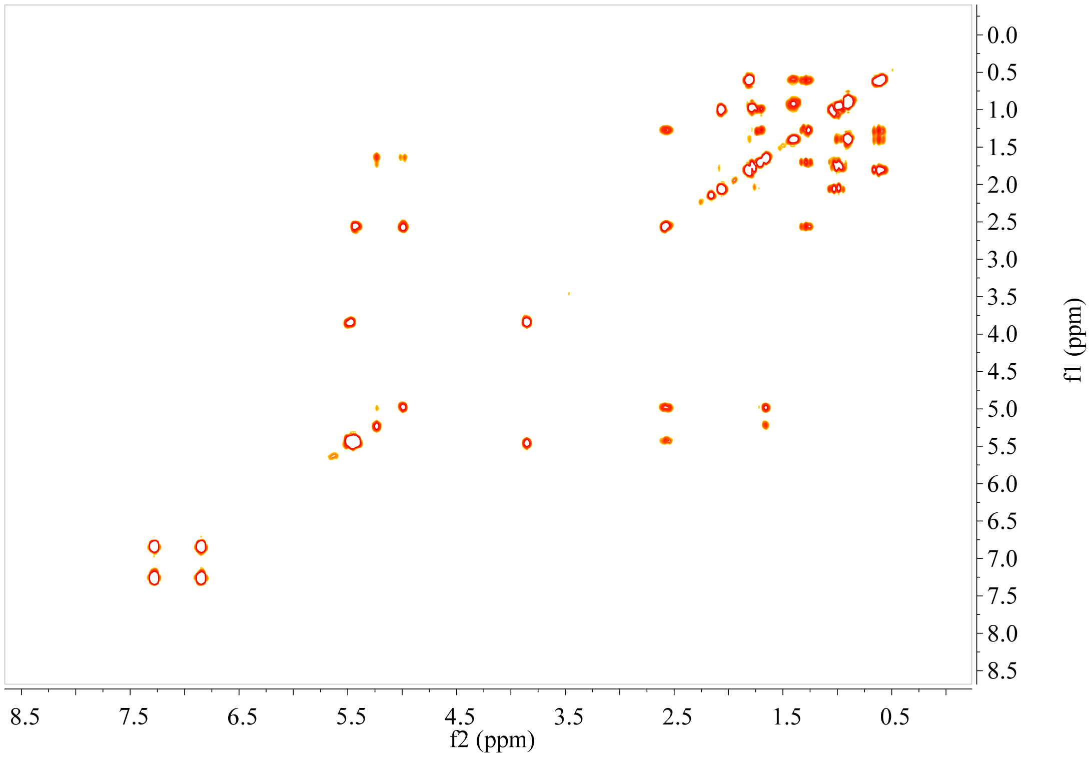


S35: Edited HSQC spectrum (500 MHz CD3CN) of hydroxyl-ilicicolin H

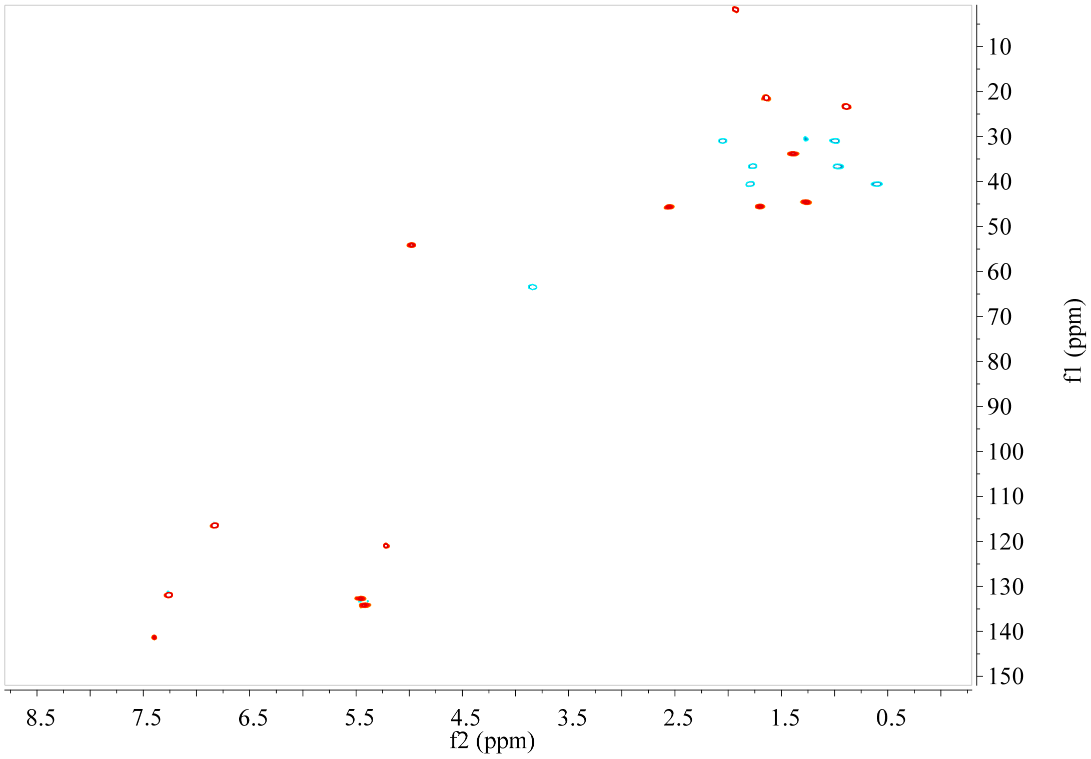

S36: HMBC spectrum (500 MHz $CD_3CN$) of hydroxyl-ilicicolin H

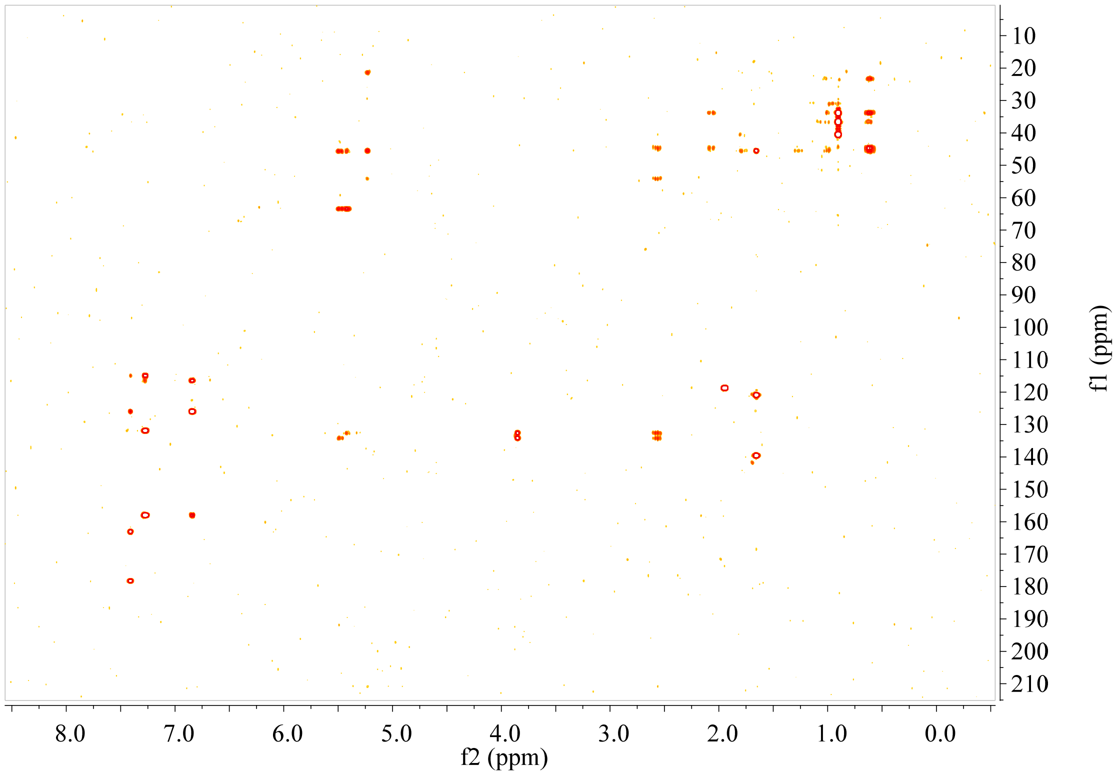


S37: NOESY spectrum (500 MHz $CD_3CN$) of hydroxyl-ilicicolin H

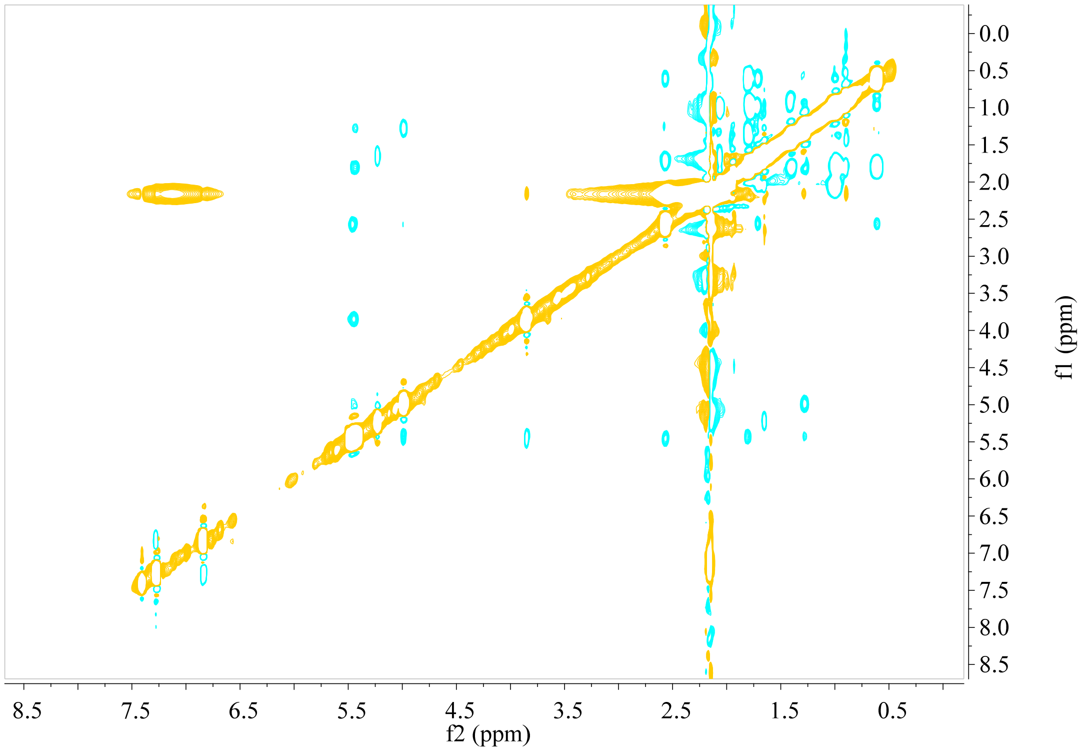

S38: $^{1}$H-NMR (800 MHz CD$_3$CN) of ilicicolin I

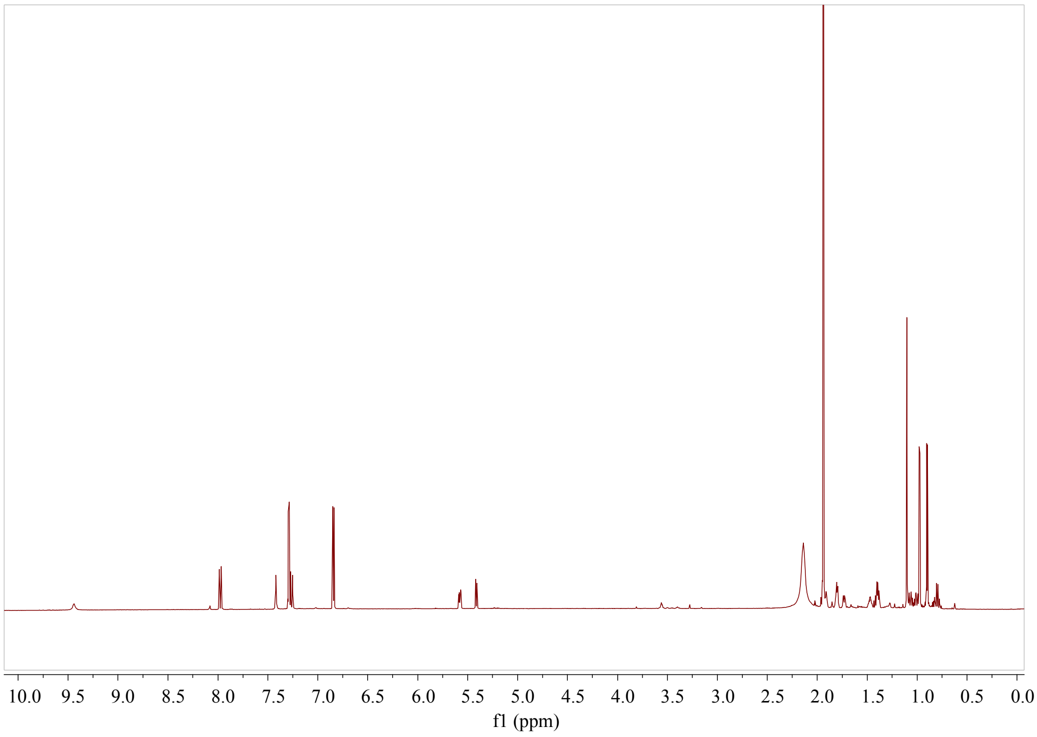


S39: $^{13}$C-NMR (800 MHz CD$_3$CN) of ilicicolin I

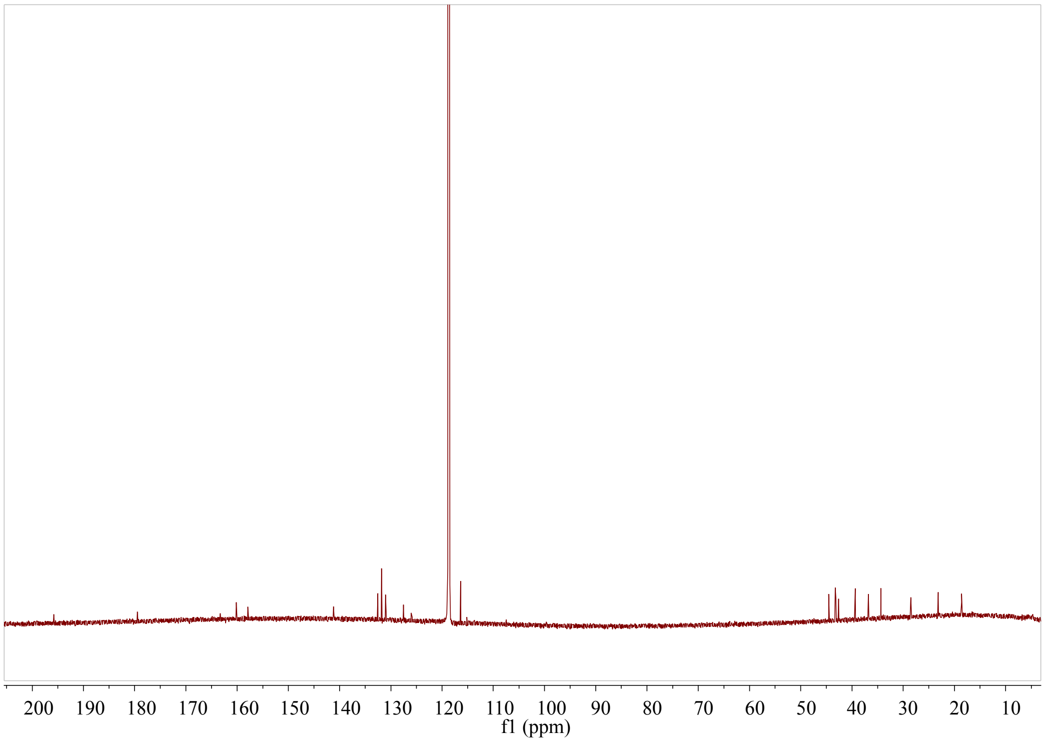

S40: COSY spectrum (800 MHz $CD_3CN$) of ilicicolin I

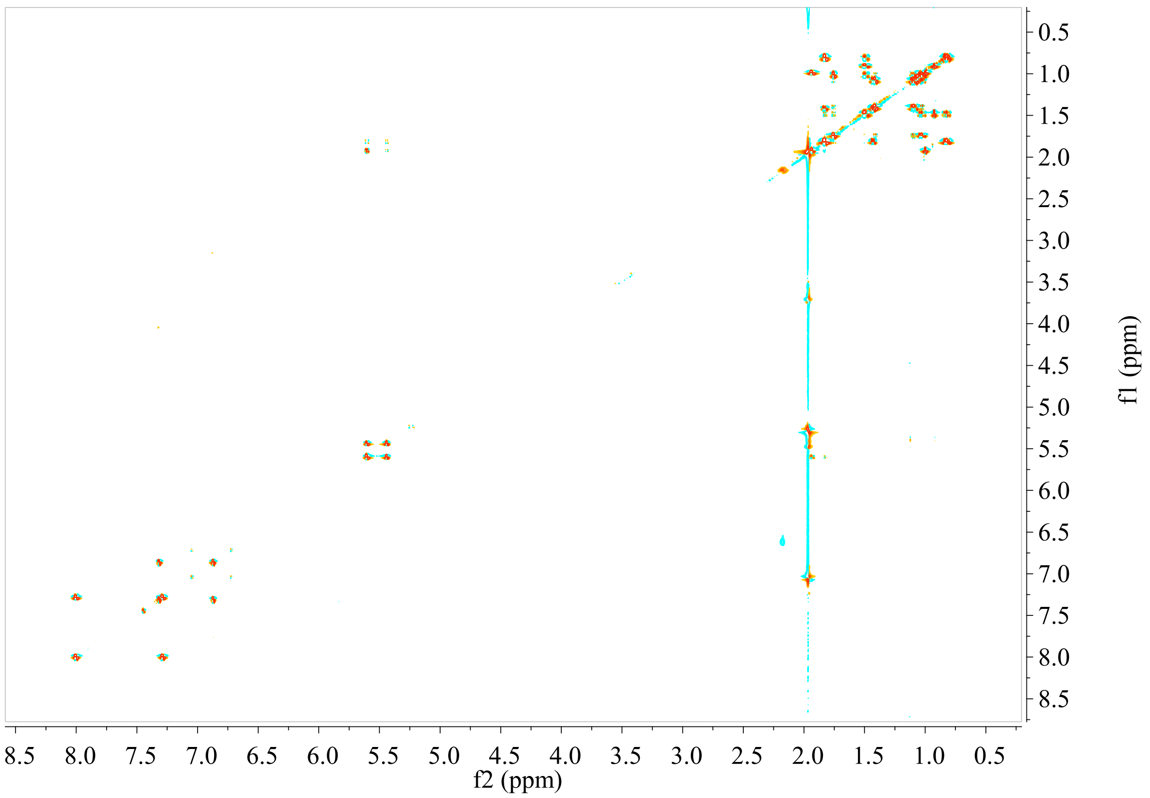


S41: HSQC spectrum (800 MHz $CD_3CN$) of ilicicolin I

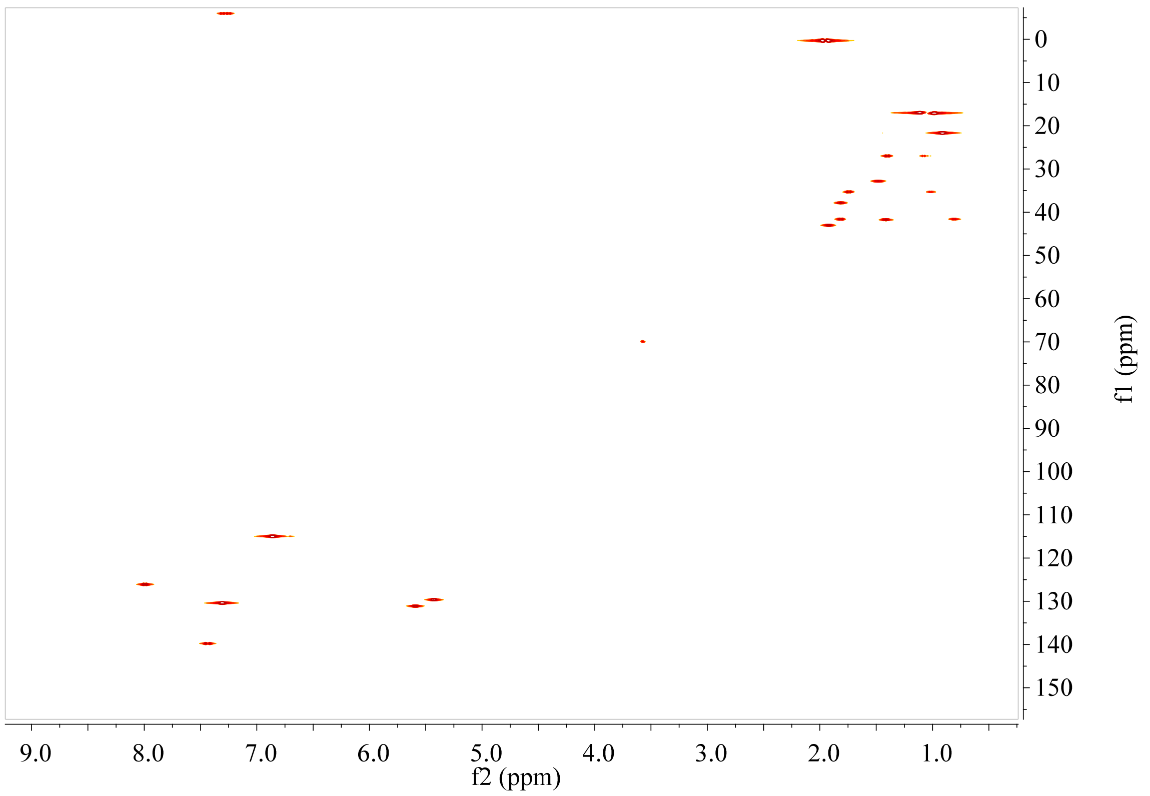

S42: HMBC spectrum (800 MHz $CD_3CN$) of ilicicolin I

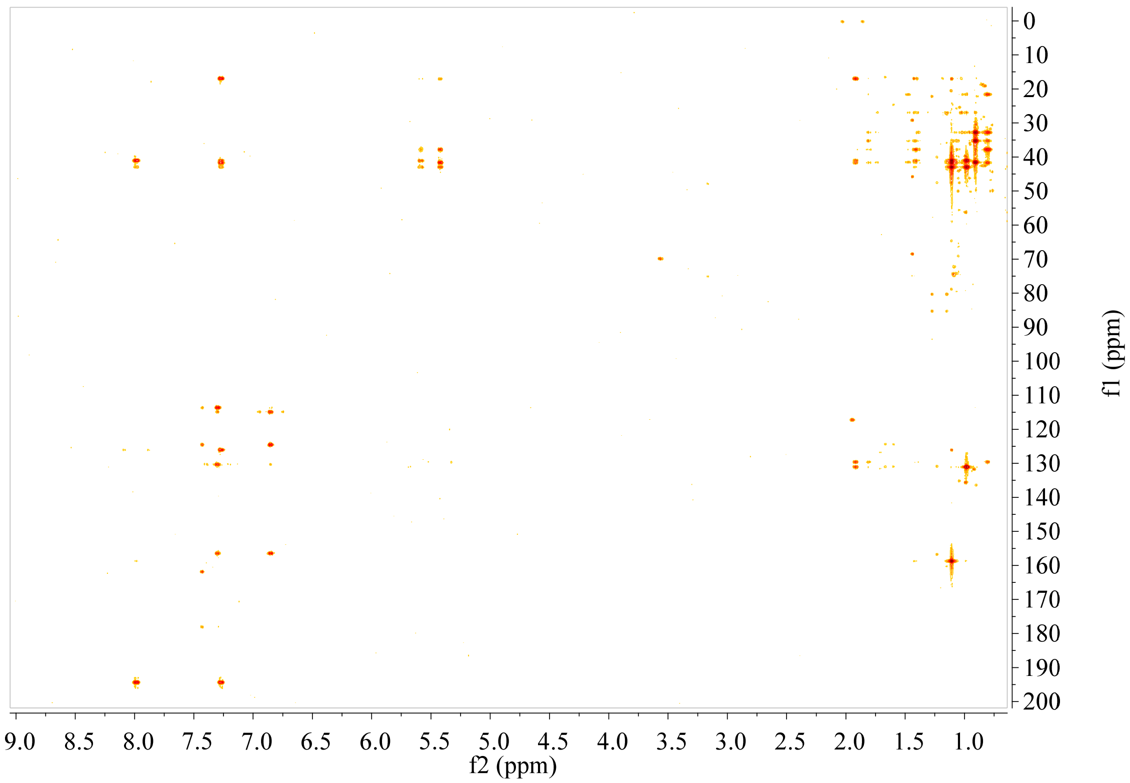


S43: NOESY spectrum (800 MHz $CD_3CN$) of ilicicolin I

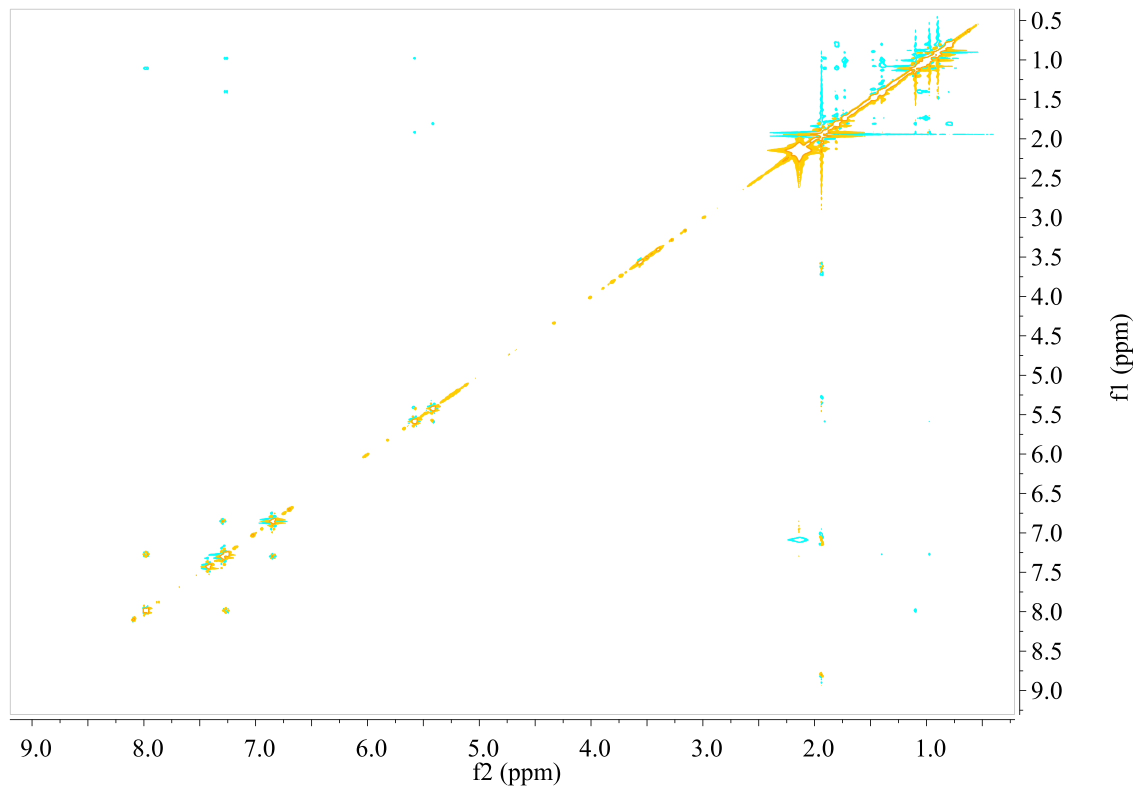

S44: $^1$H-NMR (800 MHz $CD_3CN$) of ilicicolin H

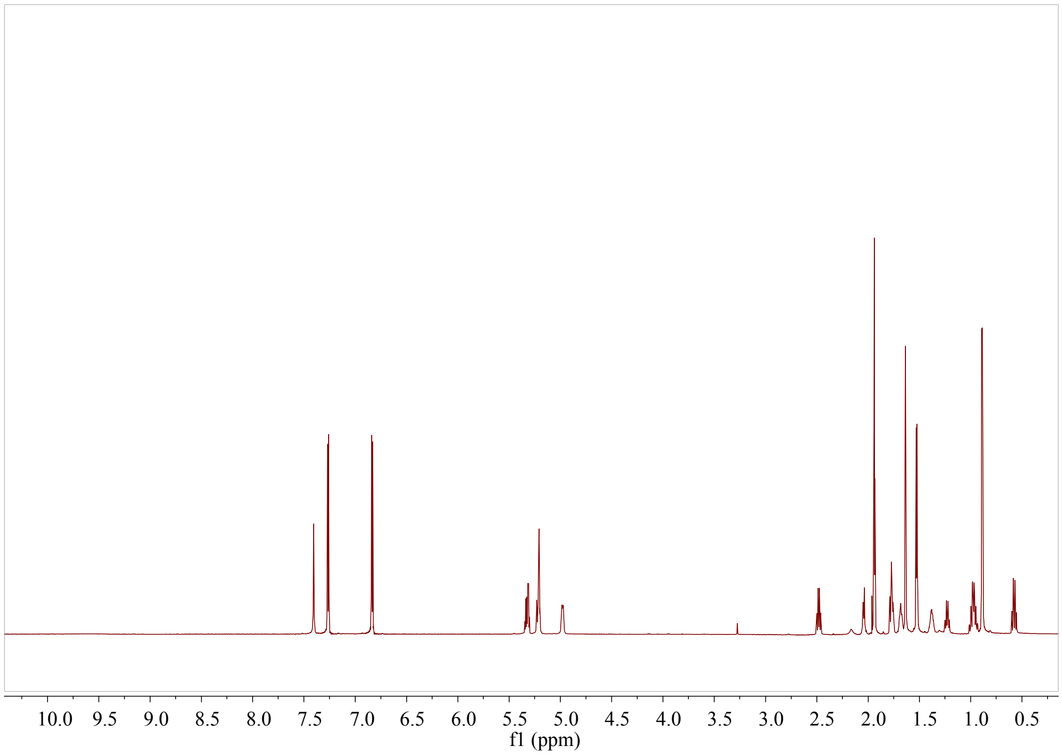


S45: $^{13}$C-NMR (800 MHz $CD_3CN$) of ilicicolin H

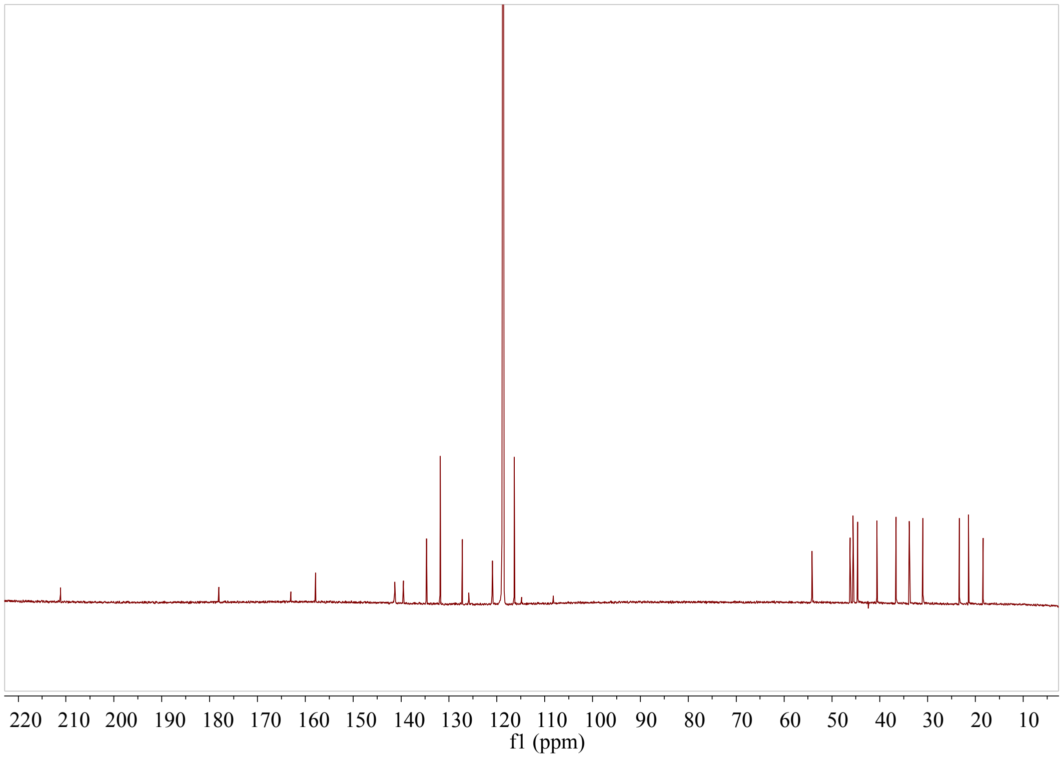

S46: Table 3. Anticancer activity of pimarane-diterpenes. $IC_{50}$ values (μM) of myrocin F, libertellenone M, libertellenone C and libertellenone E against cell lines NCH421k (glioblastoma), A549 (lung carcinoma), MCF7 (breast carcinoma), SW480 (colorectal adenocarcinoma), DU 145 (prostate carcinoma) after incubation of each compound at (0 – 300 μM) for 48 hours.

| | NCH421k | A549 | MCF7 | SW480 | DU 145 |
|---|---|---|---|---|---|
| Myrocin F | 40 | 50 | 24 | 20 | 30 |
| Libertellenone M | 18 | 75 | 49 | 110 | 270 |
| Libertellenone C | 40 | 150 | 70 | 65 | 130 |
| Libertellenone E | >300 | >300 | >300 | >300 | >300 |

S47: Dose response curves of pimarane-diterpenes in glioblastoma stem-like cells. Myrocin F (A), libertellenone M (B), libertellenone C (C) and libertellenone E (D) were incubated at various concentrations with GSC line NCH421k for 48 hours. The $IC_{50}$ is shown for each compound. (n = 3, biological replicates, error bars represent SEM).

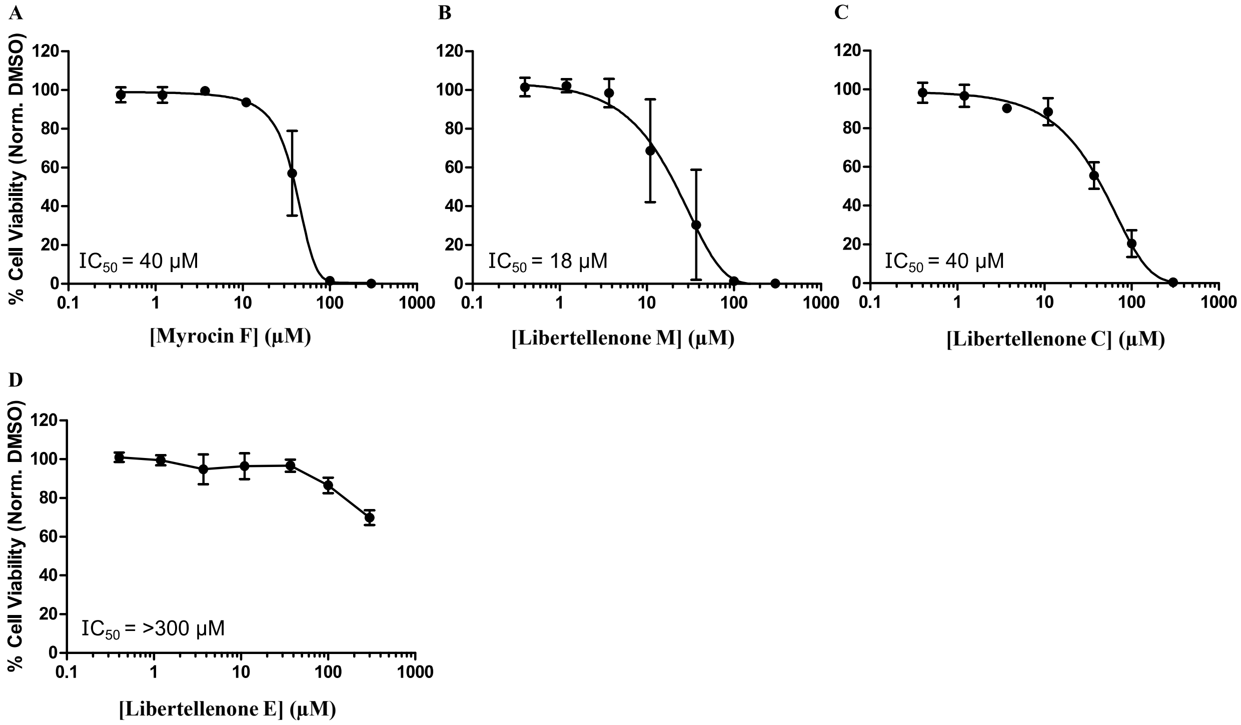

Supplement: Supplementary file 1 [file marinedrugs-15-00253-s001.pdf]
